# Biochemistry, pharmacology, and in vivo function of arginases

**DOI:** 10.1124/pharmrev.124.001271

**Published:** 2024-11-22

**Authors:** Sophia K. Heuser, Junjie Li, Silke Pudewell, Anthea LoBue, Zhixin Li, Miriam M. Cortese-Krott

**Affiliations:** 1 Myocardial Infarction Research Laboratory, Department of Cardiology, Pulmonology, and Angiology, Medical Faculty, Heinrich-Heine-University, Düsseldorf, Germany; 2 Department of Biochemistry and Molecular Biology II, Medical Faculty, Heinrich-Heine-University, Düsseldorf, Germany; 3 Cardiovascular Research Institute Düsseldorf, Düsseldorf, Germany; 4 Department of Physiology and Pharmacology, Karolinska Institutet, Stockholm, Sweden

## Abstract

The enzyme arginase catalyzes the hydrolysis of l-arginine into l-ornithine and urea. The 2 existing isoforms Arg1 and Arg2 exhibit different cellular localizations and metabolic functions. Arginase activity is crucial for nitrogen detoxification in the urea cycle, synthesis of polyamines, and control of l-arginine bioavailability and nitric oxide (NO) production. Despite significant progress in the understanding of the biochemistry and function of arginases, several open questions remain. Recent studies have revealed that the regulation and function of Arg1 and Arg2 are cell type–specific, species-specific, and profoundly different in mice and humans. The main differences are in the distribution and function of Arg1 and Arg2 in immune and erythroid cells. Contrary to what was previously thought, Arg1 activity appears to be only partially related to vascular NO signaling under homeostatic conditions in the vascular wall, but its expression is increased under disease conditions and may be targeted by treatment with arginase inhibitors. Arg2 appears to be mainly a catabolic enzyme involved in the synthesis of l-ornithine, polyamine, and l-proline but may play a putative role in blood pressure control, at least in mice. The immunosuppressive role of arginase-mediated arginine depletion is a promising target for cancer treatment. This review critically revises and discusses the biochemistry, pharmacology, and in vivo function of arginases, focusing on the insights gained from the analysis of cell-specific *Arg1* and *Arg2* knockout mice and human studies using arginase inhibitors or pegylated recombinant arginase.

**Significance Statement:**

Further basic and translational research is needed to deepen our understanding of the regulation of Arg1 and Arg2 in different cell types in consideration of their localization, species-specificity, and multiple biochemical and physiological roles. This will lead to better pharmacological strategies to target arginase activity in liver, cardiovascular, hematological, immune/infectious diseases, and cancer.

## Graphical abstract

Graphical Abstract. Biochemistry, pharmacology, and in vivo function of arginases revisited: lessons from cell-specific *Arg1* and *Arg2*^*−/−*^ mice and human studies. Arginase converts l-arginine to l-ornithine and urea and plays important roles in various biological processes, including the urea cycle in the liver, the synthesis of polyamine and l-proline, and the control of l-arginine local bioavailability for NO production in the vessel wall and inflammatory cells. Recent studies performed in cell-specific transgenic mice and in human specimens indicate that the regulation and function of Arg1 and Arg2 appear to be cell type-specific, species-specific, and profoundly different in mouse and human. Specificity and availability of arginase inhibitors in use in humans are still limited by their lack of isoform specificity and for their poor pharmacodynamic properties, which means that they have to be administered intravenously or intraperitoneally. The only approved drug so far is a pegylated arginase for the treatment of hyperarginemia related to genetic defects in the *ARG1* gene. Understanding the regulation of Arg1 and Arg2 in different cell types under consideration of their localization, species-specificity, and multiple biochemical and physiological roles could lead to better pharmacological strategies to target arginase in liver, cardiovascular, hematological, immune/infectious diseases, and cancer. OAT, ornithine aminotransferase; ODC, ornithine decarboxylase

**Figure:**
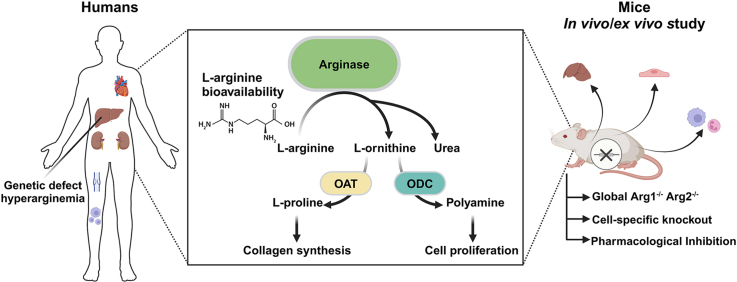

## I Introduction

**Fig. 1:**
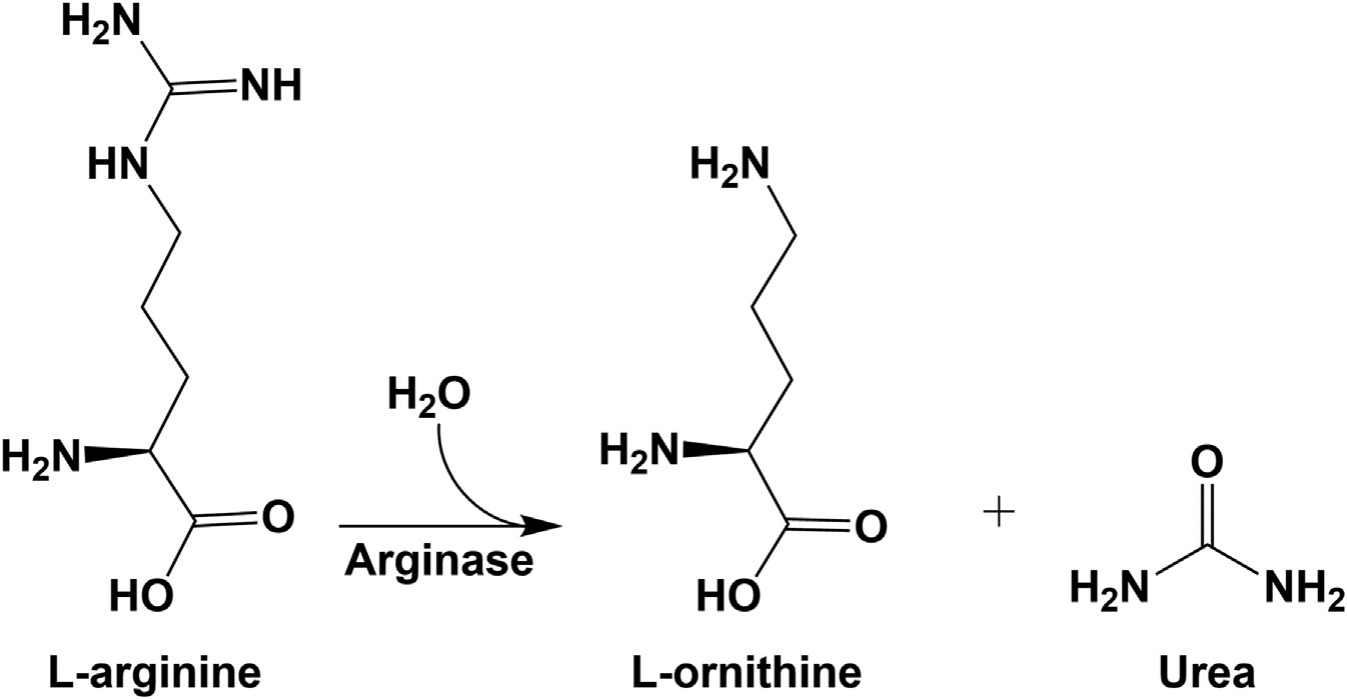
Hydrolysis of l-arginine to l-ornithine and urea catalyzed by arginase.

The enzyme arginase (l-arginine-urea hydrolase; EC 3.5.3.1) catalyzes the hydrolysis of l-arginine to l-ornithine and urea and thereby participates in the final step of the urea cycle (Fig. 1). In mammals, there are 2 isoforms of arginase, defined as arginase 1 (Arg1) and arginase 2 (Arg2), which are encoded by independent genes (*ARG1* 6q23; *ARG2* 14q24.1–24.3) (Sparkes et al, 1986; Gotoh et al, 1997). The 2 isoenzymes differ substantially in subcellular localization and function. Arg1 is mainly localized in the cytoplasm and is highly expressed in hepatocytes in the liver and participates in the urea cycle. Arg2 is a mitochondrial enzyme that was first identified in the kidney (Kaysen and Strecker 1973), but its function in the kidney and elsewhere has been unclear for many years.

Arg1 was found to regulate l-arginine bioavailability and nitric oxide (NO) synthesis from nitric oxide synthases (NOSs) in the immune system and the cardiovascular system (Durante et al, 2007); therefore, Arg1 was proposed to modulate macrophage M1/M2 polarization, suppress T cell responses, and regulate vascular endothelial function and NO-mediated cardioprotection. However, accumulating evidence obtained from cell-specific mice and multiomics analysis of human cells has demonstrated that some of these mechanisms are not modulated by Arg1 but rather by Arg2; that they depend on the cell type under consideration; that they are different in health versus disease conditions; and that they are species-specific and, in many cases, profoundly different in mouse and man.

**Fig. 2:**
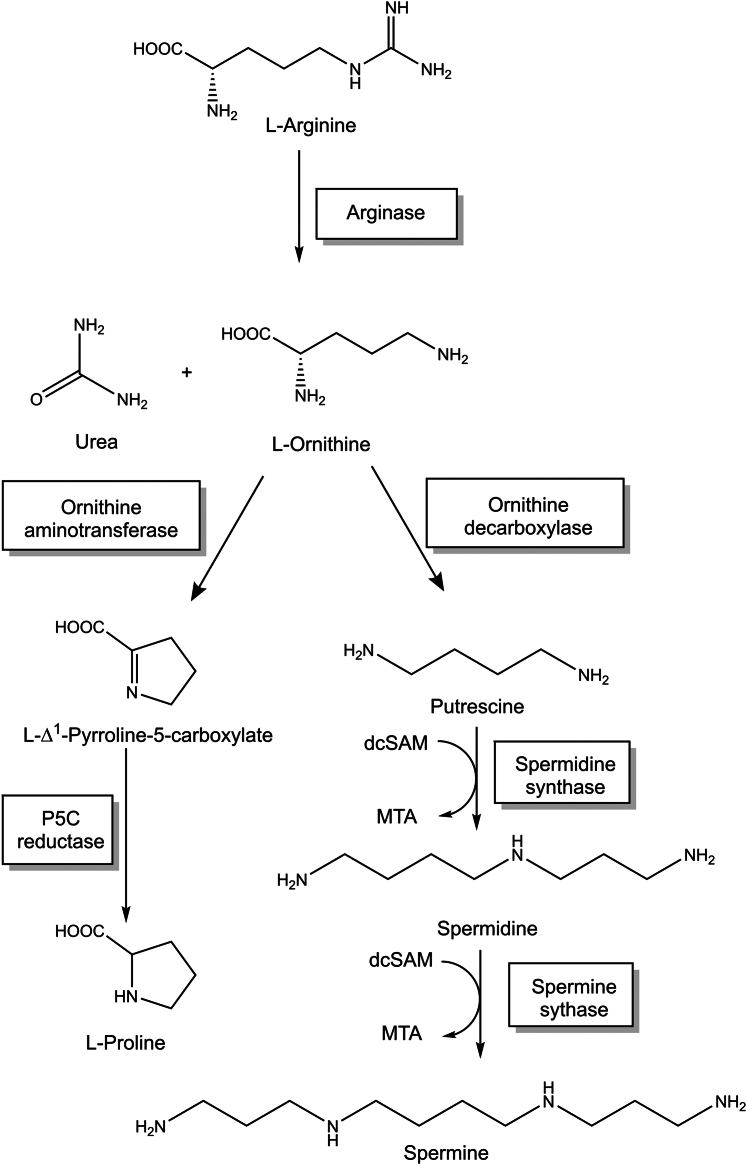
Synthesis of polyamines from l-ornithine. dcSAM, decarboxylated *S*-adenosylmethionine; MTA, 5′-methylthioadenosine.

Moreover, by synthesizing l-ornithine, arginases are also crucial for the generation and intracellular availability of polyamines, such as putrescine, spermidine, and spermine (Fig. 2). Notably, polyamines have been shown to be involved in the promotion of stem cell self-renewal (James et al, 2018), induction of autophagy (Hofer et al, 2022), protection against neurological disorders (Ghosh et al, 2020), immune cell functions, and immune suppression in the tumor microenvironment (Hayes et al, 2014).

This review summarizes the current state of knowledge on the biochemistry, pharmacology, and in vivo function of arginases, focusing on in vivo data obtained from the analysis of cell-specific Arg1 knockout (*Arg1*^*−/−*^) and Arg2 knockout (*Arg2*^*−/−*^) mice, and from human studies. Specifically, we will summarize the biochemistry, genetics, and cellular biology of arginases; their cell-specific and species-specific role in the liver, vasculature, bone marrow, blood (immune cells, red blood cells [RBCs]), and kidney; and summarize the current pharmacological strategies to target arginases, and the results of human studies using recombinant arginase and arginase inhibitors.

The careful characterization of the cell-specific role of Arg1 and Arg2 in mouse models, human cells/organoids, and human cohorts with single-cell and multiomics approaches will allow a deeper understanding of the role of arginase in specific cells and tissues, thus providing better diagnostic, prognostic, and therapeutic strategies to address cardiovascular, hematological, inflammatory, and genetic diseases related to l-arginine metabolism and cancer.

## II Biochemistry and regulation of arginase expression and activity

### A Genetic characteristics and regulation

The enzyme arginase catalyzes the hydrolysis of l-arginine to l-ornithine and urea (Fig. 1). In mammals, there are 2 isoforms of arginase, defined as Arg1 and Arg2, which are encoded by independent genes. In humans, Arg1 and Arg2 show 58% sequence homology (Morris et al, 1997, Perozich et al, 1998). The human *ARG1* gene was cloned independently by the groups of Cederbaum and Mori in the 1980s and mapped to chromosome 6q23 (Dizikes et al, 1986; Sparkes et al, 1986; Haraguchi et al, 1987). Human *ARG2* was cloned by the same groups 10 years later and mapped to chromosome 14q24.1–24.3 (Gotoh et al, 1996, 1997; Vockley et al, 1996). The gene sequence has highly conserved residues among species (Jenkinson et al, 1996) (Table 1). Structural studies identified high similarity (50%) and sequence homology in the genes encoding arginases of different species (Jenkinson et al, 1996, Perozich et al, 1998) (Table 1).

**Table 1:** Characteristics of arginases in humans, rats, mice, and zebrafish Please note that per standard nomenclature, human protein names are abbreviated with all capital letters (*ARG1* and *ARG2*), rodents with first letter capitalized (*Arg1* and *Arg2*), and zebrafish all lowercase (*arg1* and *arg2*). Gene names are italicized. In the text, we use Arg1 and Arg2 to indicate the enzymes from all species.

	Gene Name	Protein Names	Chromosome	Size	UniProt ID	Cofactors	Localization
Human	*ARG1*	Arginase 1 (ARG1) Arginase I (AI) Liver-type arginase Type I arginase	6q23.2	322 aa (35 kDa)	P05089	Two Mn^2+^ ions each Oligomerization (homotrimer)	Cytosol
		Three isoforms produced by alternative splicing (Isoform 1: 322 aa / Isoform 2: 330 aa / Isoform 3: 236 aa)					
Rat	*Arg1*	Arg1	1p12	323 aa (35 kDa)	P07824		
Mouse	*Arg1*	Arg1	10A4	323 aa (35 kDa)	Q61176		
Zebrafish	*arg1*	arg1	12	341 aa (37 kDa)	E7F8R4		
Human	*ARG2*	Arginase 2 (ARG2) Arginase II (AII) Kidney-type arginase Non-hepatic arginase Type II arginase	14q.24.1	354 aa (39 kDa)	P78540		Mitochondria
Rat	*Arg2*	Arg2	6q24	354 aa (39 kDa)	O08701		
Mouse	*Arg2*	Arg2	12C3	354 aa (39 kDa)	O08691		
Zebrafish	*ARG2*	arg2	13	347 aa (38 kDa)	Q6PH54		

Evolutionarily, it is most likely that the 2 paralogs *ARG1* and *ARG2* originated by gene duplication before amphibians and mammals diverged (Patterton and Shi, 1994; Morris et al, 1997). Among all species, arginase and other ureohydrolases share multiple conserved residues that play crucial roles in protein folding, binding of manganese ions, and substrate interaction (Kanyo et al, 1996, Perozich et al, 1998). The sequence around the catalytic site of both Arg1 and Arg2 is highly conserved, which makes it difficult to synthesize isoform-specific arginase inhibitors. Current arginase inhibitors resemble its substrate l-arginine, are mostly unselective, and display similar K_i_ or IC_50_ values (Adams et al, 2017). However, there is currently an ongoing effort to synthetize isoform-specific drugs (Gzik et al, 2024) (see also SectionV.C and Tables 3 and 4).

**Fig. 3:**
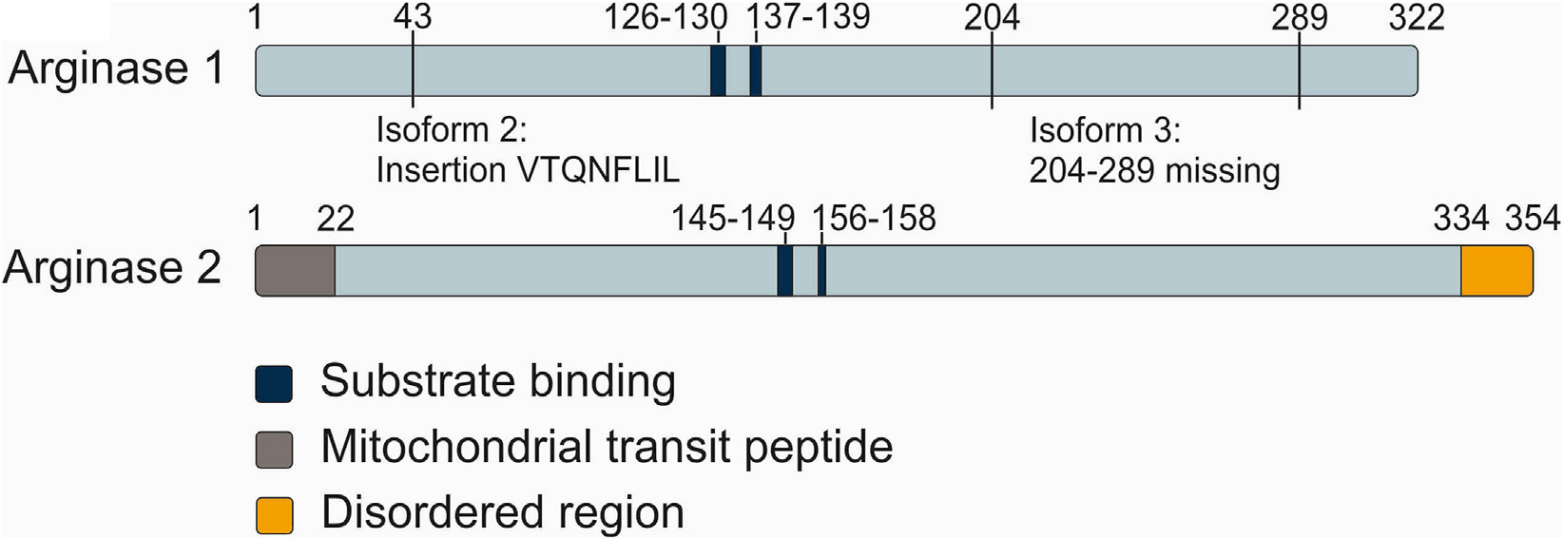
Domain organization of Arg1 and Arg2 in *Homo sapiens*. Arg1 comprises 322 amino acids (aa) and exists as the canonical protein (isoform 1), a longer variant that includes the sequence VTQNFLIL following Q43 (isoform 2), and a 204–289 aa deletion variant (isoform 3). Two substrate-binding regions (dark blue) have been identified as well as Mn^2+^-interacting aa that are not marked here. Arg2 sequence begins with a mitochondrial transit peptide (Marselli et al, 2021) followed by the main body of the enzyme (blue) and ends with a disordered region (yellow). The substrate-binding regions are marked in dark blue.

There are 3 different isoforms of the human Arg1 protein obtained by alternative splicing (Table 1; Fig. 3). The canonical isoform 1 (P05089-1) comprises 8 exons that encode 322 amino acids. Isoform 2 (P05089-2) includes an 8 amino acid insertion and therefore consists of 330 amino acids (Fig. 3). Isoform 3 (P05089-3) lacks exons 4 and 5, which encode amino acids 204–289, and therefore make a total of 236 amino acids. Only 1 sequence is known for Arg2, which is also composed of 8 exons and comprises 354 amino acids (Sayers et al, 2022).

Apart from several similarities between Arg1 and Arg2 among species, the localization, cellular function, and tissue- or cell-specific expression are rather diverse. In humans and rodents, Arg1 is constitutively expressed at high levels in the liver, particularly in the cytosol of hepatocytes, where it is responsible for catalyzing the last step of the urea cycle. This is also the reason why it was called “liver arginase” (Morris et al, 1997). The functions of Arg1, besides its role in the urea cycle, are less characterized and profoundly different in mice and man. In human, Arg1 is also expressed to a lower extent in the bone marrow, the blood, and the skin (Morris et al, 1997; Kim et al, 2002; Bruch-Gerharz et al, 2003; Munder et al, 2005). In rodents, Arg1 is constitutively expressed in multiple tissues, mainly in the liver and gastrointestinal tract, but also in the uterus and the skin (Yu et al, 2003; Choi et al, 2012).

At the cellular level, Arg1 is constitutively expressed in hepatocytes and inflammatory cells (M2 macrophages) in all species. In other cell types, including vascular endothelial cells (ECs), smooth muscle cells, and cardiomyocytes, it is expressed constitutively in a species-specific manner and in general at very low levels, but its expression is induced in disease conditions (Morris et al, 1997; Teupser et al, 2006; Gonon et al, 2012; Caldwell et al, 2015), as described in detail in the next sections. In humans, polymorphonuclear neutrophils (PMNs) constitutively express Arg1, which is localized in granules and can be released under proinflammatory conditions (Rotondo et al, 2011). Interestingly, human and primate erythrocytes constitutively express higher levels of Arg1 and display increased arginase activity in disease conditions, whereas rodent erythrocytes express Arg1 at very low levels (Spector et al, 1985).

The regulation of the expression of either isozyme is cell type-specific and can be influenced by health/disease conditions and by the presence of proinflammatory (T helper 1 [Th1]) or anti-inflammatory (T helper 2 [Th2]) cytokines. In mice, Arg1 expression was shown to be induced upon Th2 cytokine, such as interleukin (IL)-4 and -6, stimulation in M2 macrophages via signal transducer and activator of transcription (STAT) 6 signaling, by the mitogen-activated protein kinase (MAPK) - activating transcription factor-2 (ATF-2) signaling pathway, by CCAAT/enhancer binding protein (C/EBP) *β*, and by the transcription factor forkhead box O4 (FoxO4) (Gray et al, 2005; Sheldon et al, 2013; Shatanawi et al, 2015; Zhu et al, 2015; Schmok et al, 2017; Caldwell et al, 2018).

In contrast, Arg2 is mainly localized in the mitochondria and is constitutively expressed at higher levels in the kidney, bladder, and prostate but also in human skeletal muscle (Vockley et al, 1996; Morris et al, 1997; Rath et al, 2014). Lower constitutive expression of Arg2 is found in almost all cells of the body. The expression of Arg2 can be upregulated in inflammatory or disease conditions in different cell types and in a species-specific manner. For example, it was shown to be upregulated by interferon regulatory factor 3 in Jurkat cells (Grandvaux et al, 2005), by the hypoxia-inducible factor-2 in human umbilical vein ECs (Krotova et al, 2010), by activation of the ERK5 (extracellular signal-regulated kinase 5)-CREB (cyclic AMP-responsive element-binding protein) pathway in human Jurkat cells and mouse monocytes (Barra et al, 2011), and by IL-10 stimulation, likely via STAT3 activation in the mouse. It is therefore generally different from the regulation of Arg1 (Grandvaux et al, 2005; Krotova et al, 2010; Barra et al, 2011; Dowling et al, 2021).

### B Protein structure and catalysis

**Fig. 4:**
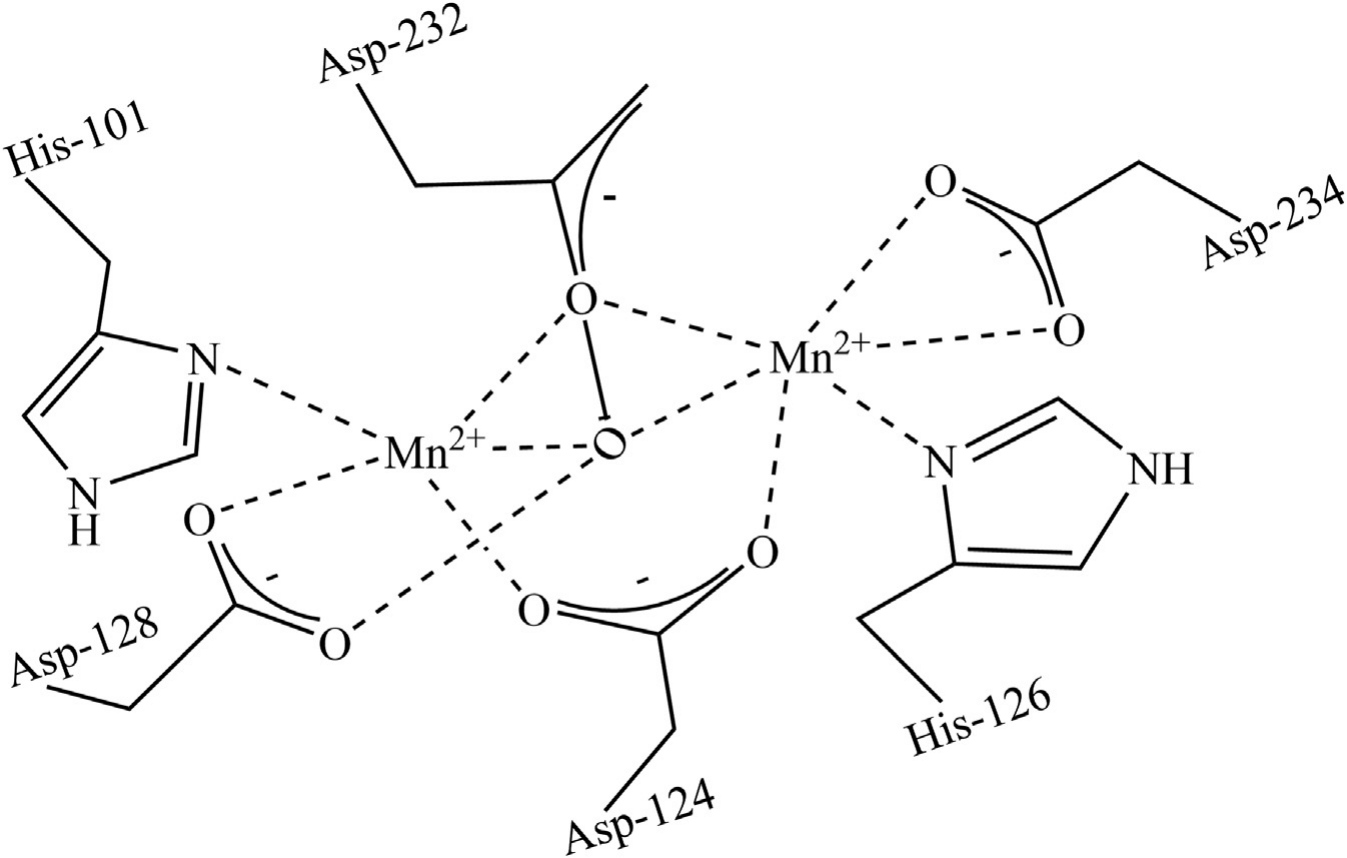
Active site of a monomer of Arg1 showing a binuclear manganese center coordinated with 2 His and 4 Asp.

Human Arg1 and Arg2 have a similar trimeric structure and catalytic mechanisms (Cama et al, 2003; Di Costanzo et al, 2005). Each monomer of arginase exhibits a typical Rossman-fold structure in which *β*-sheets are wrapped by *α*-helices (Li et al, 2022a). The molecular weight of each monomer varies among isoforms and species, ranging from 30 to 40 kDa (Table 1) (Li et al, 2022a). The crystal structure of human Arg1 was characterized by Di Costanzo et al (2005). The catalytic center of each arginase monomer contains a Mn^2+^ coordinated with 1 His and 3 Asp (Fig. 4). The spin-coupled Mn^2+^-Mn^2+^ structure is formed in the catalytic center and activates arginase (Fig. 4) (Li et al, 2022a). Mn^2+^ is required as a cofactor for converting l-arginine to l-ornithine and urea. Interestingly, if Mn^2+^ is replaced with Co^2+^, the catalytic efficiency (k_cat_/k_M_) of Arg1 is increased (Stone et al, 2010).

Interestingly, the kinetics of the hydrolysis of l-arginine to l-ornithine and urea catalyzed by isolated/recombinant arginase is dramatically different when compared to the enzyme expressed in cellular compartments. Purified rat liver arginase is characterized by a Michaelis constant (K_m_) of 1 mM and a maximum rate (V_max_) of 4380 μmol/min per mg in the presence of 10 mM MnCl_2_ at pH 7.5 (Reczkowski and Ash, 1994). In contrast, recombinant human Arg1 and Arg2 expressed in HEK293T cells are characterized by a K_m_ of 3.3 mM and V_max_ of 34 nmol/min per mg and a K_m_ of 1.9 mM and V_max_ of 883 pmol/min per mg, respectively, at physiological pH value of 7.4 (Tommasi et al, 2018). These differences need to be taken into consideration for applications of pharmaceutical preparations containing recombinant arginase, such as the pegylated-arginases, which was recently approved for treating hyperarginemia in patients with genetic deficiency of arginase (see Section V.A).

Interestingly, it was proposed that arginase kinetics in cells is modified by the presence of binding partners that regulate the catalytic activity of the enzyme. Examples of binding partners regulating arginase activity are human flotillin, which was proposed to bind Arg1 in human RBCs (Jiang et al, 2006) or the embryonic stem cell-expressed Ras, which was found to interact with Arg1 in hepatic stellate cells (HSCs) (Pudewell et al, 2022).

In addition, the concentration and availability of l-arginine may contribute to regulating the kinetic activity of arginase enzymes. For example, in cultured RAW 264.7 cells and primary murine alveolar macrophages, Arg2 or Arg1, respectively, were described to compete for l-arginine bioavailability with the inducible NO synthase (iNOS), which catalyzes the oxidation of l-arginine to l-citrulline and leads to high-output NO synthesis (Wang et al, 1995; Hey et al, 1997; Sonoki et al, 1997; Momma and Ottaviani, 2022). Therefore, Arg1 was proposed to control l-arginine bioavailability and NO production by iNOS in murine alveolar macrophages and other cell types coexpressing iNOS in proinflammatory conditions (Sonoki et al, 1997; Rath et al, 2014), including human keratinocytes (Bruch-Gerharz et al, 2003). However, the intracellular l-arginine concentrations (∼100 *μ*M) and the K_m_ of iNOS (2.8 *μ*M) are much lower than the K_m_ for Arg1 (2 mM) (Garganta and Bond, 1986; Stuehr et al, 1991).

Arg1 was also proposed to compete with endothelial NOS (eNOS) for the common substrate l-arginine in ECs. The intracellular concentration of l-arginine in ECs is ∼100 *μ*M, which should saturate eNOS; however, paradoxically, NO production by eNOS in ECs can be increased further by increasing the extracellular l-arginine concentrations. It remains unclear how excess extracellular l-arginine can increase eNOS activity. The ability of extracellular l-arginine to increase NO production in the presence of arginase was defined as “the l-arginine paradox,” and it is still partially unresolved (Girerd et al, 1990; Lundberg and Weitzberg, 2022).

### C Summary

To summarize, the 2 isoenzymes Arg1 and Arg2 catalyze the same reaction, and their tertiary and quaternary structures are similar. The kinetics of the reaction are affected by the cell type, sublocalization in the cells (cytoplasm, granula, mitochondria), the presence of a binding partner (eg, flotillin or Ras), and the availability of l-arginine for the reaction.

## III Biochemical assays for the determination of arginase activity and L-arginine metabolomics

Biochemical assays for the determination of arginase activity and new metabolomics approaches have played a crucial role in understanding arginase function and its involvement in different cellular processes. This section will discuss the various biochemical assays used to determine arginase activity and arginine bioavailability and their applications for studying its role in systemic l-arginine metabolism in humans.

### A The urea assay: Colorimetric determination of urea formation in cells and tissues

The first method to determine arginase activity was developed in 1945. The arginase activity in liver or RBCs was quantified colorimetrically as the formation of urea by derivatization with *α*-isonitrosopropiophenone (ISPF) (Archibald, 1945; Van Slyke and Archibald, 1946). Later, it was modified by others to fit a 96-well plate format and applied for determination of arginase activity in leucocytes with a detection limit of 0.02 μmol urea (Corraliza et al, 1994). The method was then further improved and adapted by other groups for other sample types (Romero et al, 2008; Yang et al, 2013; Bhatta et al, 2017; Heuser et al, 2022; Pudewell et al, 2022).

In this assay, the samples (cells or tissue lysates) are preincubated at 58–60 °C with 7–10 mM MnCl_2_ as a cofactor for the activation of arginase. Subsequently, a millimolar concentration of l-arginine (500 mM) is added into the mixture incubated for 1 minute to ≥1 hour at 37 °C according to specific arginase activity (ie, activity/milligrams of protein) expected in the specific cell/tissue of interest. The enzymatic reaction is terminated by adding an acidic mix composed of H_2_SO_4_, H_3_PO_4_, and H_2_O, v/v/v, 1:3:7 to denature the proteins in the sample and allow for the derivatization reaction, which requires acidic conditions. A solution of ISPF (9% in ethanol) is then added to the samples and incubated for at least 30–45 minutes at 100 °C. The urea concentration in the samples is then determined colorimetrically by measuring the absorbance of the pink ISPF-adduct with urea at 540 nm and quantified by comparing the absorbance of a standard curve prepared by using standard concentrations of urea. Based on this method, arginase-dependent urea production in different cells and tissues, including kidney, liver, and brain, was determined and compared among healthy and diseased conditions as well as in different species (Iwata et al, 2002; Romero et al, 2008; Bagnost et al, 2009; Bhatta et al, 2017; Heuser et al, 2022; Pudewell et al, 2022).

The main disadvantage of the urea assay for analysis of arginase activity in cells and tissues is the lower sensitivity and signal-to-noise ratio due to the presence of urea and other colored contaminants (such as heme) in biological samples. For example, in plasma the background urea concentration is very high and need to be removed by using size exclusion centrifugation filters. This procedure is however not always applicable to tissue or cell lysates, thus limiting the accuracy of the assay for samples with very low specific activity. An example of cells with low specific activity of arginase are rodent RBCs or platelets (see below Section IV.C).

### B Colorimetric determination of l-ornithine

In 1952, Chinard et al described a single cuvette assay based on the colorimetric reaction of ninhydrin reagent with l-ornithine at very low pH (Chinard, 1952). Later, the method was optimized for microplates for the detection of arginase activity by analyzing the production of l-ornithine in human hemolysates and cultured human erythroleukemic cells (K562 cells) (Iyamu et al, 2008). If applied for determination of arginase activity in cells and tissues, this method has some major disadvantages, including the interference of endogenous l-ornithine found in the sample at the steady state, as well as its complex trafficking and metabolism, which also includes the formation of downstream products such as polyamine.

### C The radioactive assay: Conversion of ^14^C-l-arginine into l-ornithine and ^14^C-urea

Rüegg and Russell first proposed the determination of arginase activity in bovine liver, calf serum, and murine macrophage extract by applying l-[guanido-^14^C]-arginine (Rüegg and Russell, 1980). Before application into the assay, the substrate l-[^14^C]-arginine was purified using an ion exchange column prepared with a Dowex 50W X8 (hydrogen form) resin, which removes potential contaminants (including spontaneously hydrolyzed arginine) and leads to a very low background. According to their method, 1 volume of glycine buffer containing 75 mM glycine, 2 mM MnCl_2_, and 0.02% thymol blue at pH 9.7 was premixed with 7 volumes of 1 M l-arginine solution containing l-[^14^C]-arginine and water (v/v, 1:6) and 10 mM MnCl_2_ and then incubated at 56 °C, pH 7.5. The reaction was terminated by adding acetic acid with 7 M urea, 10 mM l-arginine, and 0.001% methyl red at pH 4.5 to each sample. The time-dependent formation of ^14^C-urea was measured by scintillation counting at different time points from 2 to 120 minutes (Rüegg and Russell, 1980).

By using l-[guanido-^14^C]-arginine, Spector et al (1980) established a similar assay for the detection of arginase activity in erythrocyte lysate. The produced ^14^C-urea was converted into ammonia and ^14^CO_2_ by Jackbean urease, which was trapped by filter paper soaked with NaOH as Na_2_^14^CO_3_ followed by scintillation counting. By using this method, the authors compared arginase activity in RBCs of different species, including humans and primates, rats, rabbits, cats, and dogs; the arginase activity was normalized as micromoles l-arginine hydrolyzed per gram hemoglobin per hour (Spector et al, 1985). They found <1 *μ*mol urea/g hemoglobin per hour in RBCs from BALB/c mouse, rat, rabbit, cat, and dog, in contrast to >900 *μ*mol urea/g hemoglobin per hour in humans.

Further optimization of this method was carried out by Morris et al (1998). This assay was applied by many researchers to analyze arginase activity in cells, tissues, and blood, and it is still one of the most sensitive and accurate assays. By applying this assay, they found that the arginase activity in the plasma and RBCs of individuals with sickle cell disease (SCD) was significantly higher than that in healthy controls (individuals with SCD vs healthy controls, 37.7 ± 2.9 vs 23.5 ± 1.7 nmol/mg/min in plasma and 2.1 ± 2.1 vs 0.4 ± 0.2 μmol/mL per hour in RBCs) (Morris et al, 2005a).

### D Systemic analysis of l-arginine bioavailability and l-arginine metabolism

With the discovery that upregulation of arginase activity may play an important role in disease conditions, there has been a growing interest in the analysis of arginase activity and l-arginine bioavailability in human cohorts, and in preclinical studies in experimental animals. As mentioned above, l-arginine is the common substrate for arginase and the NOS enzymes; therefore, systemic l-arginine bioavailability is thought to be dependent on the relative activity of both enzyme classes, their expression, and the compartmentalization of l-arginine (Elms et al, 2013). For the analysis of l-arginine bioavailability, the “global l-arginine bioavailability ratio” (GABR) was proposed. GABR is calculated as the ratio between l-arginine level and the levels of l-ornithine and l-citrulline (GABR = l-arginine/(l-ornithine + l-citrulline)), which are metabolic products of arginase and NOS activity, respectively (Tang et al, 2009). Further studies have investigated the levels of l-arginine, l-ornithine, and l-citrulline in plasma of human cohorts (Kövamees et al, 2016b; De Santo et al, 2018; Burrage et al, 2019; Fan et al, 2021).

Alternatively, for determination of serum levels of l-arginine, l-ornithine, and l-citrulline, asymmetric dimethylarginine (ADMA), and symmetric dimethylarginine, some researchers applied an high-performance liquid chromatography method based on fluorescent derivatization with 6-aminoquinolyl-*N*-hydroxysuccinimidyl carbamate (AccQ-Fluor) (Heresztyn et al, 2004; Miyazaki et al, 2018).

l-arginine, l-ornithine and l-citrulline and the derived GABR were also measured directly in plasma of subjects with coronary artery disease (CAD) by electrospray ionization tandem mass spectrometry online with an API 365 triple quadruple mass spectrometer using ^13^C_6_-arginine as the internal standard for the quantification (Tang et al, 2009).

Metabolomics approaches were also established to investigate l-arginine metabolism and its corresponding regulation in vivo by using liquid chromatography-quadrupole time-of-flight mass spectrometry. One example is a method for the determination of 16 amino acids, amino acid derivatives, and related compounds in plasma to identify potential biomarkers in pediatric chronic kidney disease (Benito et al, 2016). The metabolites comprised l-homoarginine, l-homocysteine, l-arginine, symmetric dimethylarginine, ADMA, dimethylglycine, *S*-adenosylhomocysteine, *S*-adenosylmethionine, l-citrulline, betaine, creatine, creatinine, glutathione, methionine, glycine, and cysteine (Benito et al, 2016). Another example is the quantitative analysis of l-arginine metabolites, polyamines, and acetylated polyamines in various biological matrices such as liver, muscle, adrenal glands, and brain in mice (Langner et al, 2022). Further examples of applications of the analysis of arginase metabolites in human cohorts are discussed in Section V.B and summarized in the related Table 5.

### E Summary

To summarize, the arginase activity in cells and tissues was analyzed by measuring the production of urea by derivatization and colorimetric assay, which has limitations due to interference with endogenous urea (eg, in plasma) or heme-containing proteins absorbing in the same range of wavelengths (eg, hemoglobin in RBCs). A more accurate method is the detection of the conversion of isotope-labeled (^13^C or ^14^C) l-arginine into their enzymatic products ^13^C or ^14^C-l-ornithine/urea from arginases, or ^13^C or ^14^C-l-citrulline from NOS by scintillation or—for nonradioactive isotopes—by mass spectrometry (MS). MS has the advantage that can be applied for metabolic analysis of l-arginine metabolites on a larger scale and does not require the use of radioactive compounds.

## IV The biological role of arginase in cells and tissues

In this section, we aim to review and discuss the cell-specific role of arginase in the liver, immune system, blood, vasculature, heart and kidney and to highlight their significance in health and disease. Specifically, we will explore the diverse functions of Arg1 and Arg2 in different tissues and describe the species-specific expressional level, localization, and regulation in rodents and humans. These differences are particularly important when pharmacological therapies are tested in preclinical models. The available cell-specific mouse models and their phenotypes are summarized in Table 2.

**Table 2:** Summary of arginase specific knockout models

Mouse Model	Targeted Isoforms	Gene Targeting Strategy	Phenotype	Reference
**Effect of Genetic Modification on Recipient Strain**				

Global *Arg1*^*−/−*^	Arg1	Replacement vector consists of genomic sequences from exon 2 to exon 8, in which exon 4 was replaced by the neomycin resistance cassette (*Neo*^*R*^)	• Hyperammonaemia • Early lethal (between day 10 and 14)	Iyer et al, 2002
Global *Arg2*^*−/−*^	Arg2	Partial deletion of exons 4 and 5 of the *Arg2* gene (by Shi et al)	• Homozygous *Arg2*^*−/−*^ mice were viable • Plasma l-arginine level ↑ • Plasma norepinephrine turnover ↑ • Hypertension	Shi et al, 2001; Huynh et al, 2009
Global *Arg2*^*−/−*^	Arg2	*Arg2*^*flox/flox*^ (exons 2 and 3) *FVB/N*^*Tg(ACTB-Cre)2Mrt*^ mice	• *Arg2*^*−/−*^ mice are unable to produce intestinal mucin • *Arg2*^*−/−*^ mice became highly susceptible to experimentally induced colitis	Park et al, 2009
Inducible global *Arg1*^*−/−*^	Arg1	*Arg1*^*flox/flox*^ (exons 7 and 8) CreER^T2^	• Lethal 2 wk after tamoxifen treatment • l-citrulline and guanidinoacetic acid ↑ • l-ornithine levels ꞊ • Other amino acids ↓	Sin et al, 2013
*Arg1^+/−^ Arg2^−/−^* mice	Arg1 Arg2	*Arg1*^*−/−*^*Arg2*^*−/−*^ mice were generated by crossing *Arg1*^*+/−*^ mice (exon 4 by Iyer et al) with *Arg2*^*−/−*^ mice (exon 4, by Shi et al)	• l-arginine level ↑ • l-ornithine levels ↓ • Liver l-ornithine levels reduced to 2% with l-arginine very highly elevated	Deignan et al, 2006

**Cell-Specific Arginase Knockout Models**				

Liver-specific *Arg1*^*−/−*^	Arg1	Deletion of exons 7 and 8 of the *Arg1* gene after intraperitoneal injection of *Arg1*^*flox/flox*^ (exons 7 and 8) mice with AAV-TBG-Cre-promoter-Cre recombinase vector	• Lethal phenotype similar to the inducible *Arg1*^*−/−*^ mice phenotype • Delivery of Arg1-eGFP AAV vector prolongs lifespan	Ballantyne et al, 2016
EC/HC *Arg1*^*−/−*^	Arg1	*Arg1*^*flox/flox*^ (exon 4) Tie2-Cre deleter	• IL-4-induced polyamine production ↑	Van den Bossche et al, 2012
EC *Arg1*^*−/−*^	Arg1	*Arg1*^*flox/flox*^ (exons 7 and 8) *Cdh5-Cre/ERT2*^*pos*^	• Expression of eNOS in the aorta ↓ • l-arginine and NO bioavailability ꞊ • Vascular endothelial function in conductance and resistance arteries ꞊ • Preserved systemic hemodynamic and cardiac performance • Increased contractile response to phenylephrine in aorta rings	Heuser et al, 2022
Microglial-specific *Arg1*^*−/−*^	Arg1	*Arg1*^*flox/flox*^ (exons 7 and 8) *Cx3cr1*^*CreER*^	• No notable morphological differences • Impaired cholinergic innervation and dendritic spine maturation in the hippocampus • Deficits in long-term memory acquisition in females	Stratoulias et al, 2023

**Effect of Disease Conditions (Selected)**				

*Infectious Disease*				
Macrophage *Arg1*^*−/−*^ *Toxoplasma gonii*/tuberculosis	Arg1	*Arg*^*flox/flox*^ (exon 7 and 8) *Tie2-Cre*^*tg/−*^ *LysM-Cre*^*tg/−*^	• Arg1^−^^/^^−^^Tie2^ showed complete *Arg1* ablation in all macrophage types, Arg1^−/−^^LysM^ showed less deletion • Host survival in *T. gondii* infection ↑ • Lung bacterial load in tuberculosis infection ↓	El Kasmi et al, 2008
Asthmatic *Arg1*-deficient BM chimeric mice	Arg1	Transfer *Arg1*-deficient BM into irradiated recipient mice Asthma model: OVA-induced or *Aspergillus fumigatus*-induced	• BM-derived Arg1 is not required for baseline immune cell development and allergen-induced inflammation • BM-derived Arg1 is the main source of allergen-induced lung arginase	Niese et al, 2009
Global *Arg2*^*−/−*^ mice Infected with *Helicobacter pylori*	Arg2	Partial deletion of exons 4 and 5 of the *Arg2* gene (by Shi et al)	• Macrophages of *Arg2*^*−/−*^ mice iNOS protein levels and NO levels ↑ • Inhibition of arginase in *Arg2*^*−/−*^ mice did not have additional effects on iNOS or NO levels	Lewis et al, 2010
Global *Arg2*^*−/−*^ mice infected with *H. pylori*	Arg2	Partial deletion of exons 4 and 5 of the *Arg2* gene (by Shi et al)	• *Arg2*^*−/−*^ macrophages undergo less apoptosis • *Arg2*^*−/−*^ macrophages more abundant • *Arg2*^*−/−*^ macrophages iNOS ↑ • *Arg2*^*−/−*^ macrophages nitrotyrosine staining ↑	Lewis et al, 2011
EC and HC *Arg1*^*−/−*^ in endotoxemia	Arg1	*Arg1*^*flox/flox*^ (exon 4) *Tie2Cre*^*tg/−*^	• Inflammatory response ↑ • NO production by iNOS ↑ • Depressed microcirculatory flow in the jejunal	Wijnands et al, 2014
Double mutant *Arg2*^*−/−*^*Nos2*^*−/−*^ mice infected with *H. pylori*	Arg2	Double mutant *Arg2*^*−/−*^*Nos2*^*−/−*^ obtained by crossing *Arg2*^*−/−*^ mice (obtained by deletion of exons 4 and 5 of the *Arg2* gene) and *Nos2*^*−/−*^ mice obtained by deletion exons 12 and 13 of the *Nos2* gene	• In *Arg2*^*−/−*^, gastric polyamine synthesis and catabolism ↑ • *Arg2*^*−/−*^ and Arg2^−/−^ Nos2^−/−^, gastritis ↑ colonization ↓ • In *Arg2*^*−/−*^, M1 macrophage activation ↑, *NOS2*^*−/−*^ and *Arg2*^*−/−*^ *Nos2*^*−/−*^, M1 macrophage activation =	Hardbower et al, 2016
*Inflammatory Disease/Asthma*				
Asthmatic EC/HC *Arg1*^*−/−*^	Arg1	*Arg1*^*flox/flox*^ (exon 4) *Tie2Cre*^*tg/−*^ or *LysMCre*^*tg/−*^ Asthma model: OVA-induced allergic asthma in female mice	• *Arg1* allele was virtually completely deleted in the lungs of knockout^Tie2^ mice, but incompletely in knockout^LysM^ mice • Improved peripheral lung function in OVA-treated knockout^Tie2^ mice • Air hyperreactivity and lung inflammation was not altered in knockout^Tie2^ mice	Cloots et al, 2013
Asthmatic myeloid cell *Arg1*^*−/−*^ in female mice	Arg 1	*Arg1*^*flox/flox*^ (exon 4) *Tie2Cre*^*tg/−*^ *LysMCre*^*tg/−*^ Asthma model: OVA-induced allergic asthma in female mice	• Arg1 positive cells completely absent from the lungs of OVA-treated knockout^Tie2^ mice, but only reduced in knockout^LysM^ mice • Compared to male mice, females show more decline of arginine-metabolizing and -transporting genes, OVA-specific IgE ↓ • Methacholine responsiveness and accumulation of inflammatory cells =	Cloots et al, 2017
*Diabetes/CVD*				
Diabetic *Arg2*^*−/−*^	Arg2	Partial deletion of exons 4 and 5 of the *Arg2* gene (by Shi et al)	• Albuminuria ↓ • Macrophage recruitment ↓ • Renal blood flow ↑	Morris et al, 2011
Diabetic *Arg1*^*+/−*^*Arg 2*^*−/−*^	Arg1 Arg2	*Arg 1*^+^^*/−*^*Arg 2*^*−/−*^ mice were generated by crossing *Arg 1*^*+/−*^ mice (exon 4, by Lyer et al) with *Arg 2*^*−/−*^ mice (exon 4/5 by Shi et al) + STZ treatment	• Impairment of EC-dependent vasodilation ↓ • Tissue oxidation, vascular stiffness, and coronary fibrosis ↓	Romero et al, 2012
EC *Arg1*^*−/−*^ mice fed a high-fat/high-sucrose diet	Arg1	*Arg*^*flox/flox*^ (exon 7 and 8) *Cdh5-Cre*^*pos/neg*^	• Prevention of endothelial dysfunction	Bhatta et al, 2017
Diabetic EC/HC *Arg1*^*−/−*^	Arg1	*Arg1*^*flox/flox*^ (exon 4) *Tie2Cre*^*tg/−*^ mice diabetic model, induced by STZ treatment	• l-arginine concentration in plasma ↑ • Diabetes-induced alterations in arterial smooth muscle reactivity and endothelium-dependent relaxation ꞊	Chennupati et al, 2018
*Renal Disease*				
EC *Arg2*^*−/−*^ and proximal tubular cell *Arg2*^*−/−*^ with unilateral ureteral obstruction	Arg2	*Arg2*^*flox/flox*^ (exon 3) *Tie2-Cre*^*pos/neg*^ *Ggt1-Cre*^*pos/neg*^	• EC *Arg2*^−^*^/^*^−^, level of renal fibrosis ↓ • Proximal tubular epithelial cell Arg2 knockout, level of fibrosis ꞊	Wetzel et al, 2020
Renal tubular cells *Arg2*^*−/−*^	Arg2	*Arg2*^*flox/flox*^ (exons 3, 4, 5, and 6) Pax8-rtTA/LC1 mice	• Urea concentration and osmolality gradients along the corticomedullary axis ↓ • Tissue damage after unilateral I/R injury • Albuminuria and aminoaciduria	Ansermet et al, 2020
*Atherosclerosis*				
Erythroid *Arg1*^*−/−*^ in *apoE*^*−/−*^ background + high-fat diet	Arg1	*Arg*^*flox/flox*^ (exon 7 and 8) *apoE*^*−/−*^*EpoR-Cre*^*pos/neg*^ mice + Western diet (high cholesterol)	• Atherosclerotic lesion size at the aortic root ꞊ • Vascular NO bioactivity, smooth muscle osteoblastic differentiation, and atherosclerotic lesion calcification ↑ • l-ornithine, proline in vascular smooth muscle cells expression ↑	Gogiraju et al, 2022

AAV, adeno-associated virus; BM, bone marrow; HC, hepatocyte; OVA, ovalbumin; STZ, streptozotocin.

### A Role of arginase in the liver

The liver is composed of 4 major cell types: hepatocytes (∼70%), HSCs (∼13%), sinusoidal ECs (∼10%), and liver resident macrophages also defined as Kupffer cells (∼7%) (Racanelli and Rehermann, 2006; Chen and Tian, 2021). Interestingly, all these cells express Arg1 and are essential for maintaining liver homeostasis (MacParland et al, 2018).

**Fig. 5:**
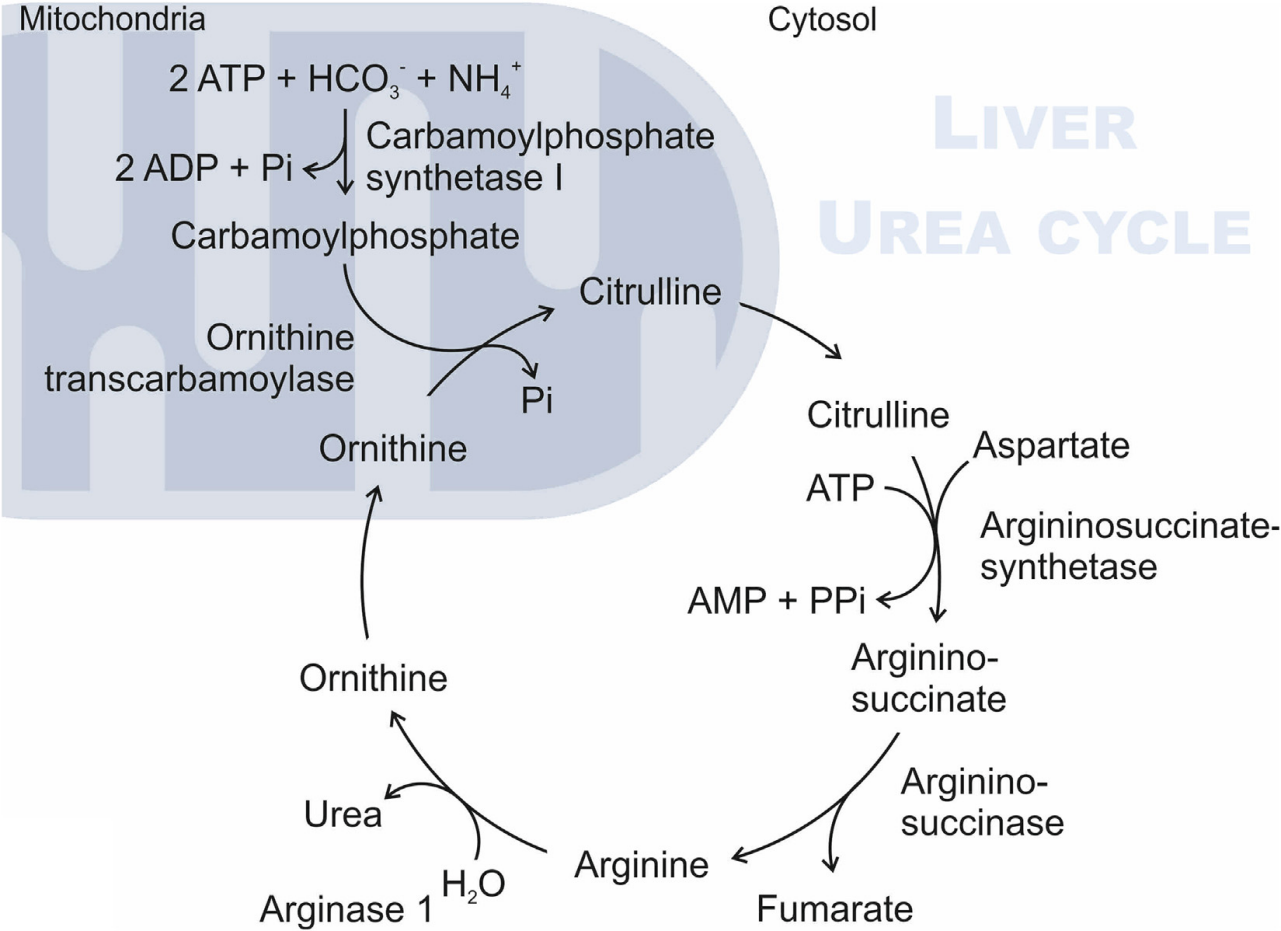
Urea cycle in the liver. The urea cycle is divided into 5 steps. The first 2 steps consist of the generation of carbamoylphosphate from ammonia and synthesis of l-citrulline, which takes place in the mitochondria. l-Citrulline is transported to the cytosol and undergoes conversion into argininosuccinate by arginosuccinate synthetase. Arginosuccinate is then cleaved into l-arginine and fumarate by argininosuccinase, and then Arg1 in the cytosol hydrolyzes l-arginine to l-ornithine and urea in a final step. l-Ornithine is transported back into the mitochondria, where it is transformed again into l-citrulline. Urea is exported into the blood and excreted in the urine via the kidney.

Arg1, the so-called liver-type arginase, is predominantly and constitutively expressed in the liver, especially in hepatocytes (MacParland et al, 2018). The main role of arginase in the liver is the detoxification of ammonia from the body, which is produced as a result of the catabolism of amino acids and is potentially neurotoxic. Ammonia is detoxified in the urea cycle by forming urea, which can be then excreted by the kidney (Krebs and Henseleit, 1932; Strong et al, 2021) (Fig. 5).

The urea cycle can be divided into 5 enzymatic steps. The first 2 steps are located in the mitochondria and the last 3 in the cytoplasm of hepatocytes: (1) ammonium, carbon dioxide, and 2 ATPs are converted into carbamoylphosphate by carbamoylphosphate synthetase I; (2) carbamoylphosphate is combined with l-ornithine to l-citrulline; (3) l-citrulline and aspartate are condensed to argininosuccinate by argininosuccinate synthetase using 1 molecule of ATP; (4) argininosuccinate is cleaved into l-arginine and fumarate by argininosuccinase; and finally (5) Arg1 hydrolyzes l-arginine into l-ornithine and urea (Pelley, 2012; Barmore et al, 2024). Urea is exported from the hepatocytes into the blood toward the kidney, where it is excreted in the urine. This also explains the high concentrations of urea found in plasma, which limits the use of the urea assay for this compartment (see SectionIII.A).

The transport of L-ornithine and L-citrulline in and out of the mitochondria is carried out by l-ornithine carrier 1 and 2 and solute carrier family 25 member A29 (SLC25A29) (Fiermonte et al, 2003; Porcelli et al, 2014). However, the expression of SLC25A29 is lower that of other transporters. Therefore, the role of SLC25A29 in the urea cycle is likely limited (Camacho and Rioseco-Camacho, 2009).

Besides the urea cycle, l-arginine can also be consumed by eNOS or iNOS to produce l-citrulline and NO in liver ECs (Poisson et al, 2017) but also in hepatocytes, HSCs, and Kupffer cells/macrophages (Cunningham and Porat-Shliom, 2021; Pudewell et al, 2022). Furthermore, in the liver, l-arginine can be converted into agmatine or creatine, whereas l-ornithine can be further catabolized into polyamines, l-proline, or glutamate. The urea cycle and its intermediates are tightly regulated (Wu and Morris, 1998). In individuals carrying a genetic mutation of liver Arg1, the levels of l-arginine in plasma are elevated (Diez-Fernandez et al, 2018), indicating that the higher arginase activity in the liver limits the export of liver l-arginine into the plasma. In line with those findings, global constitutive *Arg1*^*−/−*^ mice display hyperarginemia and die between day 10 and 14 after birth because of hyperammonemia (Iyer et al, 2002).

Conditional mouse models of Arg1 deficiency (*Arg1*^*flox/flox*^ mice) were generated by targeting exons 7 and 8 (El Kasmi et al, 2008) or exon 4 (Van den Bossche et al, 2012) (Table 2). Late-onset global *Arg1*^*−/−*^ mice and hepatocyte-specific *Arg1*^*−/−*^ mice showed a similar phenotype characterized by hyperarginemia, hyperammonemia, and dysregulation of amino acid metabolism, but without any increase in l-ornithine (Kasten et al, 2013; Sin et al, 2013). Interestingly, the same phenotype was found in another study in hepatocyte-specific *Arg1*^*−/−*^ mice (Ballantyne et al, 2016), demonstrating that Arg1 in hepatocytes plays a major role in regulating the systemic levels of ammonia and l-arginine. Other pathways that metabolize l-arginine, such as NO production by eNOS or iNOS, are mainly influenced by the extracellular l-arginine concentration (MacKenzie and Wadsworth, 2003; Shin et al, 2011).

Polyamines play an important role in liver regeneration and homeostasis. The levels of polyamines are regulated by arginase levels, import and export of polyamines and amino acids, as well as the expression of the rate-limiting enzyme l-ornithine decarboxylase, which converts l-ornithine to putrescine (Luk, 1986; Dayoub et al, 2006; Uemura and Gerner, 2011; Okumura et al, 2016; Sagar et al, 2021). Recently, it was shown that Arg1 plays a major role in the maintenance of quiescence of HSCs, suggesting an effect of downstream polyamine synthesis (Pudewell et al, 2022). The detailed role of arginase in sinusoidal liver ECs is less known. The consequence of EC-Arg1 knockout in mice was not specifically studied in the liver in detail. However, mice lacking EC-Arg1 did not show a specific liver phenotype (at least in our hands) (Heuser et al, 2022), and other investigators did not mention any liver phenotype in similar models (Table 2) (Bhatta et al, 2017). Arg1 is also expressed in resident liver macrophages (or Kupffer cells) in mouse liver. Although the canonical role of arginase in mouse bone marrow and alveolar macrophages has been well studied (see Section IV B), cell-specific analysis of the effects of arginase in Kupffer cells on liver pathophysiology is still lacking.

### B Role of arginase in the immune system

Arginases are crucially involved in various aspects of inflammation and immunomodulation both in health and disease conditions. Increased arginase activity has been involved in inflammation-triggered immune dysfunction, tumor immune escape, fibrosis, immunosuppression, and immunopathology of infectious diseases (Bronte and Zanovello, 2005; Munder, 2009; Murray, 2016; Martí and Reith, 2021). The regulatory role of Arg1 and Arg2 in the immune response profoundly differs between mice and humans.

**Fig. 6:**
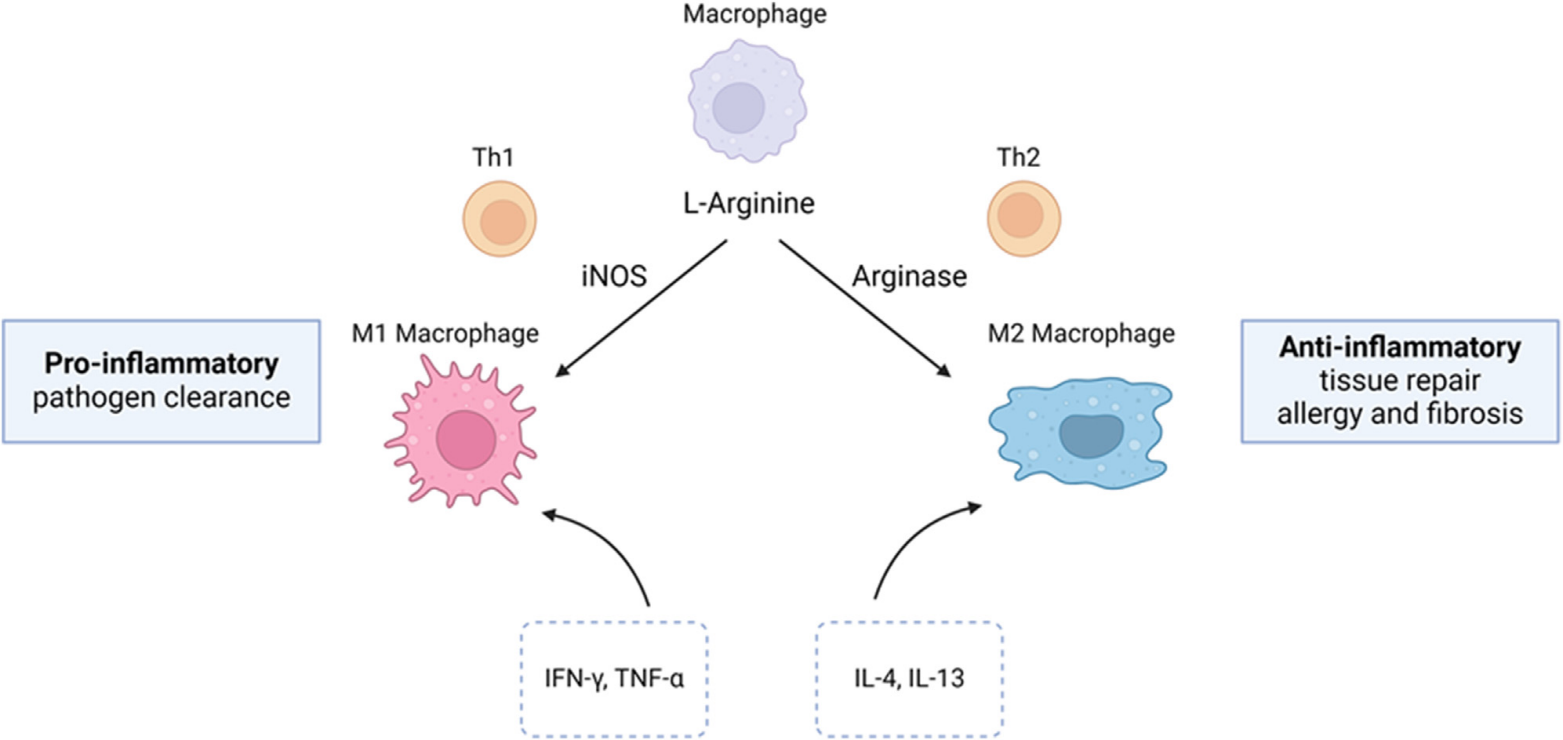
Arginase/iNOS pathway in mouse macrophages. IFN-γ, interferon gamma; IL, interleukin; iNOS, inducible nitric oxide synthase; Th, T helper; TNF-α, tumor necrosis factor alpha. Figure created with BioRender.com.

According to a classical view, mouse macrophages can be classified as belonging to 2 subtypes named M1 and M2 based on their role in the inflammatory response and their expression of iNOS (M1) or Arg1 (M2) (Fig. 6) (Thomas and Mattila, 2014; Murray, 2017). In this model, the balance between arginase and iNOS activity in macrophages dictates the outcome of immune responses. M1 macrophages preferentially metabolize l-arginine via iNOS into NO and l-citrulline and orchestrate the first proinflammatory phase of the immune response; in contrast, M2 macrophages metabolize l-arginine via Arg1 into l-ornithine and urea and are mainly involved in anti-inflammatory responses (Thomas and Mattila, 2014; Murray, 2017). The underlying mechanism involves the activation of iNOS expression in M1 macrophages by Th1-derived cytokines (IL-1-*β*, tumor necrosis factor-*α*), and interferon-*γ* induces iNOS expression via activation of transcription factors such as NF*κ*B and AP1 and drives the classical M1 activation pathway. Th2 cytokines such as IL-4, IL-10, and IL-13 suppress iNOS activity and promote Arg1 expression (Bronte and Zanovello, 2005; Thomas and Mattila, 2014; Martí and Reith, 2021). Recently, it was shown that Arg2 is present in the mitochondria of proinflammatory M1 macrophages and is essential for IL-10-mediated metabolic downregulation, promoting the resolution of inflammation (Dowling et al, 2021). Another possibility is that the cross-talk between M1 macrophages expressing iNOS and M2 macrophages expressing Arg 1 may occur via control of l-arginine bioavalability and transport via CAT1 and CAT2; however this needs to be verified experimentally.

In humans, the M1/M2 dichotomy is not well defined and is controversially discussed in the literature (Munder, 2009; Thomas and Mattila, 2014). In human blood, circulating monocytes do not express Arg1; instead, human Arg1 is constitutively expressed in PMN granules, is released in response to proinflammatory stimuli, and regulates immune T cell responses (Munder et al, 2005; Munder et al, 2006; Oberlies et al, 2009). Less is known about the role of Arg1 in human tissue macrophages and—in general—about the role of Arg2 in these cells, except that Arg2 is constitutively expressed in mitochondria and contributes to l-arginine metabolism (Martí and Reith, 2021).

T lymphocytes play a central role in the adaptive immune response. It is well known that l-arginine starvation impairs T cell functions by multiple mechanisms (Geiger et al, 2016; Martí and Reith, 2021). T cell proliferation is dose-dependent on l-arginine, with maximal proliferation occurring at plasma concentrations ∼100 *μ*mol/L (Ochoa et al, 2001). CD8^+^ T cells show a more pronounced dependency on l-arginine availability than CD4^+^ T cells. Moreover, dietary l-arginine supplementation improves thymic weight and T cell reactivity in both rats and humans (Martí and Reith, 2021).

Mechanistically, l-arginine starvation impairs T cell function through the downregulation of the CD3*ζ* subunit of the T cell receptor (TCR) complex, crucial for TCR assembly and activation (Munder et al, 2006; Munder, 2009). Additionally, l-arginine deprivation disrupts TCR signaling, reduces IL-2 production, and affects cell cycle regulators, causing T cell arrest in the G0-G1 phase (Martí and Reith, 2021). l-arginine starvation also inhibits glycolysis in T cells without affecting mitochondrial function, although high l-arginine levels can enhance CD8^+^ T cell antitumor activity in vivo (Grzywa et al, 2020).

Human T cells constitutively express mitochondrial Arg2 (Lowe et al, 2019, Martí i Líndez et al, 2019), while the constitutive expression of Arg1 is under debate (Murray, 2016; Martí and Reith, 2021). Therefore Arg2 regulates T cell intracellular l-arginine metabolism and plays a critical role in T cell function (Martí and Reith, 2021). Inhibition or deletion of Arg2 enhances T cell activation and antitumor responses, independent of extracellular l-arginine levels (Grzywa et al, 2020; Martí and Reith, 2021). Arg2 also supports regulatory T cell function and survival, indicating its potential as a therapeutic target in autoimmune and neoplastic diseases (Grzywa et al, 2020). Based on the observations that arginase inhibits T cell in the tumor microenvironment and thus promot cancer growth, novel immunotherapy vaccines targeting Arg1 or Arg2 have been developed and are now undergoing clinical trials (Martinenaite et al, 2019; Weis-Banke et al, 2020; Lorentzen et al, 2022; Niu et al, 2022).

Human granulocyte subpopulations and especially PMNs express both isoforms of arginase. In these cells, Arg1 is not found in the cytoplasm but rather in the cytoplasmic granules and, as briefly mentioned previously, Arg1 release in the extracellular space exerts immunosuppressive functions by depletion of l-arginine and inhibition of T effector cell responses (Munder et al, 2005; Munder et al, 2006; Oberlies et al, 2009). Surprisingly, the expression of Arg1 is not regulated by Th2 cytokines and other stimuli in these cells (Munder, 2009; Murray, 2016). Similar T cell immunosuppressive activity of arginases is found in myeloid-derived suppressor cells (MDSCs). MDSCs are a heterogeneous population of immature myeloid cells at different stages of myelopoiesis exerting immunosuppressive function through their ability to metabolize and deplete l-arginine, which is needed for T cell-mediated responses (Bronte et al, 2016; Ostrand-Rosenberg and Fenselau, 2018). Indeed, MDSCs express high levels of both Arg1 and iNOS (Gabrilovich and Nagaraj, 2009) and were described to inhibit T cells by high-output NO production (Jia et al, 2010) and by inducing l-arginine starvation of T cells (Raber et al, 2014). Arg1 is also crucial for the inhibition of allo-stimulated T cell by MDSCs (Bronte et al, 2003). Hence, arginases and in general l-arginine metabolic enzymes in MDSCs are considered to be excellent molecular targets of immunoregulatory compounds in infectious diseases and cancer (Table 3).

**Table 3:** Established and potential therapeutic applications

Therapeutic Intervention	Indication	Status	Reference
Pegylated arginase 1	1. Arg1 deficiency 2. Arginase auxotrophic tumors 3. Immunosuppression	1. Approved as orphan drug by the EMA 2. Open-label phase 2 trial 3. Not tested	1. Russo et al, 2024 2. Cheng et al, 2021 3. Munder, 2009
l-Arginine/l-citrulline supplementation	1. Chest pain 2. T2DM 3. Endothelia dysfunction	1. Clinical trial 2. Clinical trial 3. Clinical trial	1. Lerman et al, 1998 2. Shatanawi et al, 2020 3. Chin-Dusting et al, 1996
Arginase inhibitor	1. T2DM (+ CVD) 2. Advanced/metastatic solid tumors with upregulation of Arg1 3. Infection with parasites 4. Pulmonary hypertension in SCD	1. Clinical tests/phase 1 clinical trial 2. Phase 1 clinical trial 3. Mouse studies 4. Mouse studies	1. Shemyakin et al, 2012; Kövamees et al, 2014 2. Steggerda et al, 2017; Kuboki et al, 2024; Naing et al, 2024 3. Li et al, 2022b 4. Morris et al, 2005a; Steppan et al, 2016

EMA, European Medicines Agency.

In the tumor microenvironment, abundant arginase activity is mainly related to the presence of MDSCs, and l-arginine metabolism is one of the metabolic pathways responsible for tumor progression (Kim et al, 2018). Moreover, upregulation of either Arg1 or Arg2 expression/activity has been reported in several cancer types (Graboń et al, 2009; de Boniface et al, 2012; Bedoya et al, 2014). Accumulating research indicates that inhibiting the potent immunosuppressive mechanisms of MDSCs can be a therapeutic target to restore T cell activity and immunotherapy success in antifungal therapy (Law et al, 2020). Indeed, pharmacological inhibition of MDSC-derived Arg1 expression by either SB202190, which is a specific inhibitor of p38, or vandetanib, an orally administered receptor tyrosine kinase inhibitor, significantly enhanced T cell-mediated antifungal responses against *C. neoformans* infection (Li et al, 2022b). It has also been reported recently that by using the arginase inhibitor OAT-1746, the negative effects of Arg1 in ovarian carcinoma can be mitigated (Czystowska-Kuzmicz et al, 2019). Please refer also to Section V.C and Tables 3 and 4.

**Table 4:** Arginase inhibitors used in human-based in vitro/ex vivo studies

Name	Structure	Test	US National Clinical Trial Number	Administration	Application	Reference
**First Generation**						

ABH (2-(*S*)-amino-6-boronohexanoic acid)		In vitro	n.a.	n.a.	n.a.	Van Zandt et al, 2019
BEC (*S*-(2-boronoethyl)-l-cysteine)		In vitro/ex vivo/clinical trial	n.a.	Intradermal microdialysis in combination with nor-NOHA	CVD	Busnel et al, 2005; Holowatz et al, 2006
nor-NOHA (Nω-hydroxy-nor-arginine)		In vitro/ex vivo/clinical trial	NCT 02009527 NCT 05536934 NCT 02687152 NCT 05806502	Intrabrachial infusion/ sublingual perfusion/ intradermal microdialysis/intra-arterial infusion	CVD, T2DM, obesity	Kövamees et al, 2014; Van Zandt et al, 2019

**Second Generation**						

ABH analogs (synthesized based on Ugi reaction)		In vitro/in vivo	n.a.	n.a.	n.a.	Golebiowski et al, 2013

**Third generation**						

NED 3238		In vitro	n.a.	n.a.	n.a.	Van Zandt et al, 2019
INCB001158 (formerly named CB-1158)		Clinical trial phase 1/phase 2/in vitro	NCT 03314935 NCT 02903914 NCT 03910530 NCT 03361228 NCT 03837509	Oral application	Solid tumors	Steggerda et al, 2017; Kuboki et al, 2024; Naing et al, 2024

n.a., not applicable.

In conclusion, arginases significantly influence immune responses and inflammation, with elevated activity linked to various pathological conditions such as immune dysfunction, tumor progression, and immunosuppression. The roles of Arg1 and Arg2 differ between mice and humans, particularly regarding their modulation of macrophage and T cell functions. The use of pharmacological inhibitors to improve immunotherapy outcomes or recombinant arginase to induce cancer cell l-arginine starvation shows great potential in cancer therapy.

### C Role of arginase in red blood cells

It has long been known that Arg1 is present in circulating RBCs and that its protein level differs considerably among species (Azizi et al, 1970; Spector et al, 1983). Humans and primates have high levels/activity of arginase, whereas rodents, cats, and dogs have rather low arginase activity (which is often under the detection limits of common methods) (Azizi et al, 1970; Spector et al, 1983).

In general, the proteome of circulating RBCs is made of proteins that were synthesized during their maturation from hematopoietic stem cells to proerythroblasts to reticulocytes in the bone marrow. Human erythroid cells express both Arg1 and Arg2 (Kim et al, 2002; Grzywa et al, 2021a). In human erythroid cells, the expression of arginases starts during the late phase of erythropoiesis, when the hematopoietic stem cells differentiate into proerythroblasts, while the highest protein level is found in circulating human RBCs (Grzywa et al, 2021a,b). Importantly, Arg2 was found to be upregulated 12-fold during erythroid differentiation and remained elevated in late-stage erythroblasts, whereas Arg1 was upregulated at very late-stage terminal differentiation (Grzywa et al, 2021a,b). Arg2 is probably lost when the mitochondria and the nucleus are extruded (Grzywa et al, 2021a,b). In human erythroid cells, there is also an alternatively spliced variant of Arg1 with preserved activity (Kim et al, 2002).

The strong induction of Arg1 and Arg2 in human proerythroblasts was associated with a continuous requirement for extracellular l-arginine throughout the erythroid differentiation process. Notably, l-arginine in this context was not required for the synthesis of creatine or NO, but rather for polyamine biosynthesis and hypusination of the eIF5A transcription factor (Shima et al, 2006; Gonzalez-Menendez et al, 2023). Interestingly, mouse proerythroblasts express Arg1 at lower levels than human proerythroblasts (Grzywa et al, 2021b; Shahbaz et al, 2021).

There is compelling evidence that Arg1 levels in human RBCs are increased in people with SCD (Iyamu et al, 2005; Morris et al, 2005a). SCD is caused by a range of mutations in the *β*-globin chain of hemoglobin. Hypoxia induces sickling of RBCs due to polymerization of abnormal hemoglobin (Pauling et al, 1949). Sickle cells are stiffer, more fragile, more prone to rupture, and show increased arginase activity (Iyamu et al, 2005). Specifically, it was proposed that the release of RBC protein content due to cell rupture/damage induces an increase of free hemoglobin and Arg1 in plasma. The increase in free hemoglobin in plasma leads to systemic oxidative stress and NO scavenging, while the increased arginase activity in plasma leads to reduced l-arginine bioavailability. Therefore, both free hemoglobin and arginase in plasma may contribute to the pathophysiology of SCD by promoting endothelial dysfunction and pulmonary hypertension. (Morris et al, 2005a). A similar pathophysiology was also observed in hemolytic uremic syndrome (Friberg et al, 2024).

Interestingly, other investigators proposed that liberation of arginase from human RBCs into the plasma may also exert immunosuppressive effects by l-arginine depletion (Bernard et al, 2008; Munder, 2009). This immunosuppressive effect of arginase released from RBCs could also be a possible explanation for the increased risk of invasive bacterial infection in humans with SCD.

Another role attributed to Arg1 in RBCs is the control of systemic NO bioavailability and NO release from RBCs (Yang et al, 2013). We and others have shown that eNOS is present in RBCs (Kleinbongard et al, 2006; Cortese-Krott et al, 2012). In line with this finding, Yang et al (2013) showed that the inhibition of Arg1 in human RBCs regulates eNOS-dependent export of NO metabolites and contributes to cardioprotection in a Langendorff bioassay. By comparing the cardiovascular hemodynamics and the outcome of acute myocardial infarction in RBC- and EC-specific *eNOS*^*−/−*^ mice, we recently demonstrated that eNOS present in RBCs regulates blood pressure and the levels of circulating NO metabolites and is cardioprotective (Leo et al, 2021; Cortese-Krott et al, 2022). Recently, an erythroid cell targeted *Arg1*^*−/−*^ mouse was generated by using mice expressing a Cre-recombinase under the control of the promoter for erythropoietin receptor and crossed into an *apoE*^*−/−*^ background. These mice showed increased vascular calcification on high-fat diet and increased *S*-nitrosoglutathione reductase activity in their vessels (Gogiraju et al, 2022).

It is important to point out that mice and rats are unlikely to be good models for studying the role of Arg1 in RBCs in vivo. According to all studies investigating arginase activity by monitoring the conversion of isotopically labeled l-arginine into l-ornithine and urea, the arginase activity in mouse and rat RBCs is very low or even undetectable under some conditions (Azizi et al, 1970; Spector et al, 1983). Moreover, the expression and activity of arginase in mouse monocytes is very high (Munder et al, 2005), which may contaminate RBC samples. In addition, when considering the results of mouse studies obtained with the *loxP*/*Cre* system, the specificity of the promoter and its regulation often determine the quality of the results. As mentioned previously, the *Tie2* promoter drives gene expression in both ECs and cells of the myeloid cell lineage (leukocytes) (Payne et al, 2018), and the phenotype may derive from vascular or immune cell dysfunction.

There is no doubt however that an increase in arginase activity in human RBCs has an important pathophysiological role. Independent human studies found that the levels of l-arginine in human RBCs correlated with the levels of Arg1 expression and activity in RBCs (Spector et al, 1985; Morris et al, 2000, 2005a). As previously mentioned, RBC arginase activity was found to be elevated in hematological diseases (especially in diseases caused by genetic mutations of genes codifying for hemoglobin chains like SCD) as well as cardiovascular disease (CVD) (Azizi et al, 1970; Iyamu et al, 2005; Morris et al, 2005b).

In the late 80s, Cederbaum et al proposed that the absence of arginase in the RBCs from lower animals, and its presence in the RBC from primates and humans may be the result of an evolutionary adaptation, rather than the “vestigial presence of an arcane function” (Spector et al, 1985). It is unclear whether the presence of arginase expression in RBCs confers any obvious advantage or disadvantage to the animal carrying it. This interesting perspective was sadly not pursued further.

To summarize, while human RBCs carry high levels of Arg1, mice and rats express Arg1 at a very low level; the reason of this discrepancy is unknown. An increase in RBC arginase activity plays a major role in SCD pathophysiology and was also proposed to be immunomodulatory. More studies with human erythroid precursor cells and human cohorts are required to understand the pathophysiological role of arginase in RBCs and how its levels and activity may be modulated under disease conditions.

### D Role of arginase in the vasculature

In the vasculature, l-arginine is mainly converted into NO by eNOS (EC:1.14.13.39) expressed in ECs. NO is involved in the modulation of endothelial function, vascular tone, organ perfusion, and blood pressure (Moncada et al, 1991; Farah et al, 2018; Ostrand-Rosenberg and Fenselau, 2018; Lundberg and Weitzberg, 2022). Reduced bioavailability of NO results in endothelial dysfunction and promotes hypertension, atherosclerosis, and myocardial infarction.

**Fig. 7:**
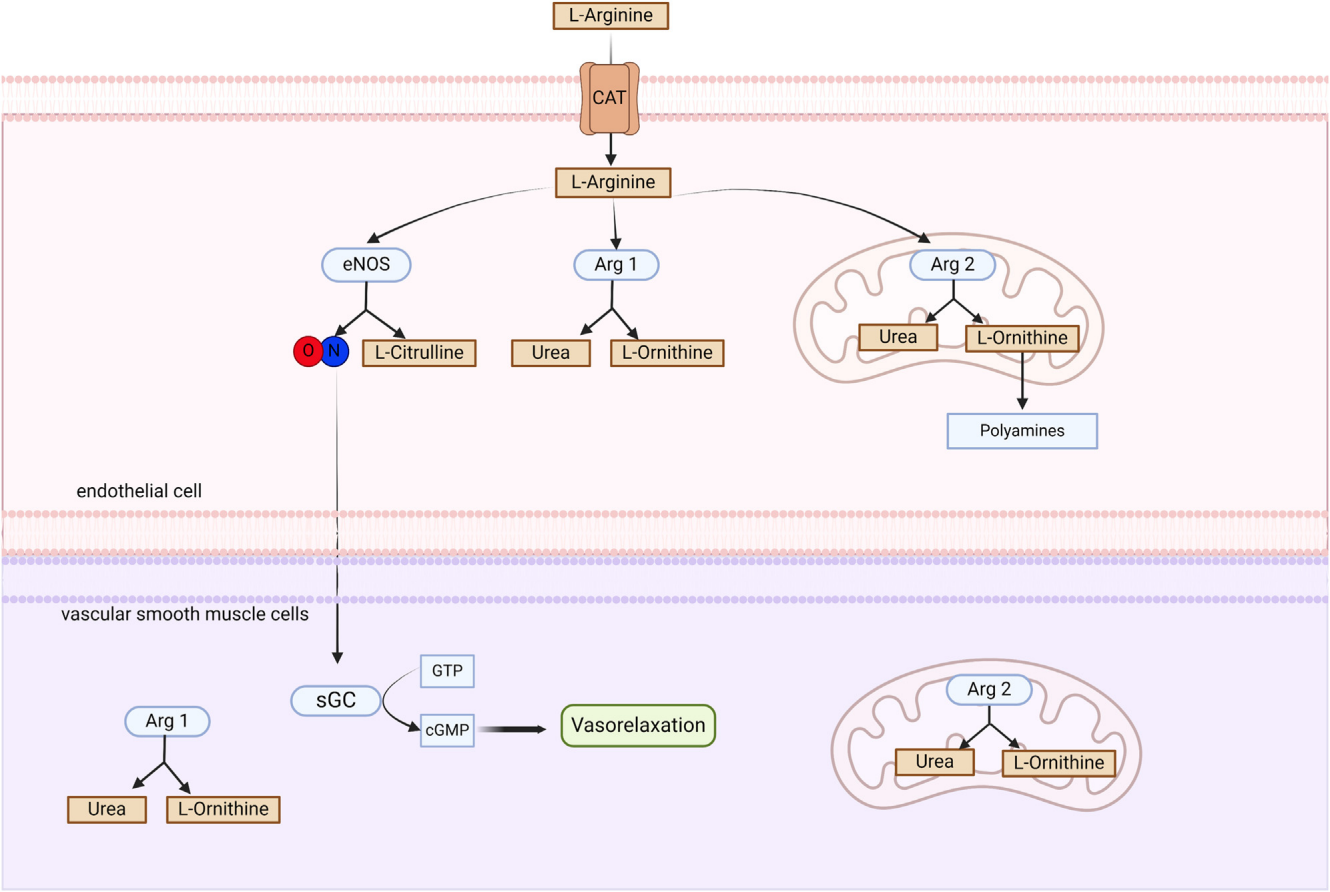
L-Arginine metabolism in ECs. In ECs, l-arginine serves as a key substrate for both nitric oxide (NO) synthesis by endothelial nitric oxide synthase (eNOS) and catabolism by Arginase 1 (Arg1) and Arginase 2 (Arg2). eNOS is well known to produce NO, which diffuses to vascular smooth muscle cells where it activates soluble guanylate cyclase (sGC), leading to vasorelaxation. Because of the coexpression of both arginase isoforms within the endothelium, they are proposed to act as functional counterparts to eNOS, indirectly regulating its activity. Data from cell-specific mouse models reveal that this competition becomes relevant only in disease conditions leading to an increase in arginase activity and is less relevant under homeostatic conditions. CAT, cationic amino acid transporter; cGMP, cyclic guanosine monophosphate; GTP, guanosine triphosphate. Figure created with BioRender.com.

In the vascular wall, ECs and vascular smooth muscle cells may express both isoforms of arginase (although there are some species-specific patterns for Arg1 or Arg2, as carotid porcine ECs, for example, express Arg2 and not Arg1 [Thacher et al, 2010]) (Fig. 7). Nevertheless, multiple studies describe arginase as a counterpart of eNOS in vascular ECs for modulating endothelial function (Kim et al, 2009; Chung et al, 2014; Krause et al, 2015). Thus, increased arginase activity in the vessel wall was proposed to limit the bioavailability of l-arginine for eNOS and therefore decrease NO production, resulting in endothelial dysfunction and hypertension (Zhang et al, 2001; Toque et al, 2013; Caldwell et al, 2018; Mahdi et al, 2020a; Li et al, 2022c).

Multiple preclinical studies in rodents showed that the oral administration of arginase inhibitors, such as 2(*S*)-amino-6-boronohexanoic acid (ABH) and *N*^*ω*^-hydroxy-nor-arginine (nor-NOHA) improved eNOS-dependent vasorelaxation and endothelial function and decreased blood pressure in spontaneously hypertensive rats, old rats, or rats fed a high-fat diet (Kim et al, 2009; Bagnost et al, 2010; Chung et al, 2014).

A similar phenotype was observed in EC-specific *Arg1*^*−/−*^ mice generated using cadherin-5 (*Cdh5*)-promoter *Cre* recombinase mice and fed a high-fat and high-sucrose diet. In these mice, the deletion of *Arg1* in ECs protected mice from vascular dysfunction (Bhatta et al, 2017; Yao et al, 2017). Interestingly, EC/myeloid cell-specific *Arg1*^*−/−*^ mice generated using *Tie2*-*Cre* recombinase mice did not improve vasomotor function in diabetic mice (Chennupati et al, 2018). It is important to mention that the *Tie2* promoter drives *Cre* recombinase expression in all subtypes of ECs, but *Cre* recombinase expression was also found in the hematopoietic cell lineage or, depending on the gene construct, in the heart valves (Payne et al, 2018). The *Cdh5* promoter drives the expression of *Cre* recombinase specifically in ECs, and it is generally considered the most specific promoter, in particular when the activity of the *Cre* recombinase is inducible by tamoxifen (Cdh5ET2-Cre mice); however, in some models in which *Cdh5*-*Cre* recombinase expression is constitutive, the expression of *Cre* recombinase was also observed in hematopoietic cells and the cardiac valve.

Work from our laboratory demonstrated that under homeostatic conditions, EC *Arg1*^*−/−*^ mice (tamoxifen-inducible Cdh5ET2-Cre) show a downregulation of eNOS in the aorta and a fully preserved vascular function and NO metabolites under basal conditions (Heuser et al, 2022). We also observed a compensatory upregulation of Arg1 in the aorta, which points to an upregulation of Arg1 in vascular smooth muscle cells (Heuser et al, 2022). Therefore, the relationship between eNOS and Arg1 in ECs in vivo is far more complex than a competition for their common substrate. Accordingly, a recent study provided quantitative evidence that in murine macrophages and human umbilical artery ECs, there was no direct competition between Arg1 and the NOS enzymes if a constant flux of l-arginine is provided (Momma and Ottaviani, 2022).

The role of Arg2 in vessels is less known. Arg2 expression in the mitochondria of ECs and smooth muscle cells is lower than that of Arg1, and their functions are difficult to discern without genetic manipulation. There are few studies investigating the role of mitochondrial Arg2 in the endothelium in mice. One animal study showed that Arg2 is the key isoform responsible for the total arginase activity in the aorta of aging mice, leading to eNOS uncoupling and endothelial dysfunction (Shin et al, 2012). This finding is supported by another study showing that mice overexpressing Arg2 in the endothelium showed endothelial dysfunction, hypertension, and enhanced atherosclerosis (Vaisman et al, 2012). In addition, Arg2 is reported to promote a proinflammatory effect, contributing to insulin resistance and atherogenesis (Ming et al, 2012; Yang and Ming, 2014). Interestingly, in porcine carotid ECs, Arg2 was upregulated by oscillatory shear stress; as a result, porcine carotid arteries subjected to oscillatory shear stress showed a decreased bradykinin-induced vasorelaxation, which could be recovered by treatment with the arginase inhibitor nor-NOHA (Thacher et al, 2010). Interestingly, porcine carotid arteries and their cellular components (ECs and smooth muscle cells) express Arg2, but not Arg1, showing a further species-specific feature of arginases in the vasculature.

Overall, these results indicate that Arg1 does not appear to be involved in the regulation of NO-dependent vasorelaxation in homeostatic conditions and that an increase in arginase activity is correlated with regulation of vascular remodeling and stiffening, probably via l-arginine depletion and synthesis of polyamines.

Further studies are needed to understand whether and how arginase expression or activity in the vascular endothelium is regulated by pathophysiological stimuli such as shear stress or turbulent flow and how this regulation is coordinated with eNOS activity.

### E Role of arginase in the heart

In the heart, Arg1 is expressed in coronary ECs and cardiomyocytes in a species-specific manner. The expression of Arg1 has been found to be upregulated in coronary arterioles in humans with type 2 diabetes mellitus (T2DM) and in homogenates of samples of the right atrial appendage collected during cardiac surgery (Chen et al, 2006; Beleznai et al, 2011). Arg1 is constitutively expressed in the cardiomyocytes of felines and affects cardiomyocyte NO signaling, whereas Arg2 is not constitutively expressed in feline cardiomyocytes (Jung et al, 2006). Rat heart lysate shows expression of both Arg1 and Arg2, but cardiomyocytes from rats express only Arg2 (Steppan et al, 2006).

Nor-NOHA-mediated arginase inhibition during ischemia-reperfusion (I/R) injury in rats resulted in reduced infarct size and elevated plasma nitrite levels in vivo (Jung et al, 2010; Tratsiakovich et al, 2013). Furthermore, Arg1 expression was significantly increased in the ischemic myocardium of rats (Jung et al, 2010). Whether these effects are caused by the expression of Arg1 in the myocardial cells (cardiomyocytes, vascular cells) or from infiltrating neutrophils or other blood cells was not further investigated. Indeed, infiltrating neutrophils are known to contribute to the infarct size, at least in rat (Williams et al, 1994). It has also been shown that in pigs, Arg1 expressed in coronary arterioles modulates NO-mediated vasorelaxation (Zhang et al, 2001). Furthermore, it has been proposed that coronary EC dysfunction plays a role in the microvascular injury occurring after I/R injury. This hypothesis is supported by the finding that mice overexpressing tumor necrosis factor-*α* show an increase in arginase activity as well as in the expression of Arg1 in ECs at basal conditions and after I/R injury (Gao et al, 2007). In addition, these mice show a significant reduction in maximal vasodilation after I/R injury as well as a decrease in eNOS expression in coronary arterioles.

To summarize, also in the heart, arginases show a species-specific and cell-specific expression. It appears that Arg1 plays a role in the pathophysiology of myocardial infarction whereas Arg2 plays an immunosuppressive and protective role, at least in rodents. The data on the expression/activity and function of arginases in human heart tissue are still too sparse to make a clear conclusion about its role in the heart. More research is needed in this direction, perhaps by using novel single-cell sequencing and mapping in heart biopsies.

### F l-Arginine metabolism and role of arginase in the kidney

**Fig. 8:**
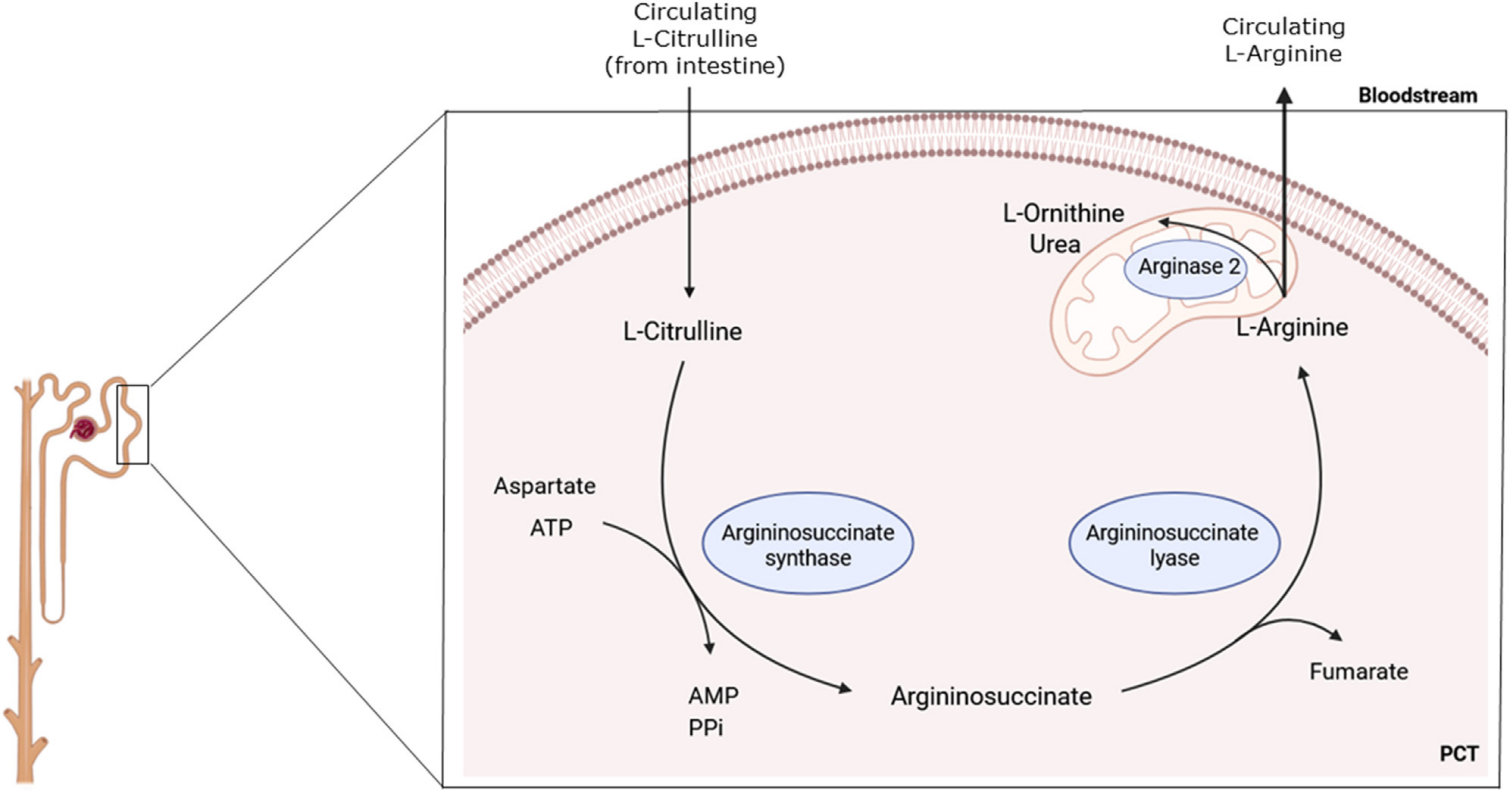
Synthesis of l-arginine in the kidney. l-Arginine is endogenously synthesized in the kidney in a reaction catalyzed by argininosuccinate synthetase and argininosuccinate lyase by using circulating l-citrulline produced in the intestine. Synthesis occurs throughout the whole length of the proximal tubule but is particularly high in the early part closest to the glomerulus, the proximal convoluted tubule (PCT), and gradually decreases in the terminal part in the outer medulla, the proximal straight tubule (PST). Only a small part of the l-arginine synthesized in the kidney is used for the production of polyamines and creatine from l-ornithine, while most of it is released into the circulation, making it available for other tissues. Figure created with BioRender.com.

The kidney plays an essential role in the endogenous synthesis of l-arginine. l-Arginine is synthesized from l-citrulline by arginosuccinate synthetase and arginosuccinate lyase (Szepesi et al, 1970; Morris et al, 1989). As mentioned in Section IV.A, there is a high turnover of l-arginine through the urea cycle in the liver; however, the urea cycle is tightly regulated such that l-arginine is promptly metabolized further and thereby does not contribute to the circulating levels of l-arginine under homeostatic conditions. In contrast, in the kidney, only a small part of the synthesized l-arginine is used for the production of polyamines and creatine from l-ornithine, while most of it is released into the circulation, making it available for other tissues (Rogers et al, 1972) (Fig. 8).

l-Arginine is synthesized throughout the length of the proximal tubule by arginosuccinate synthetase and arginosuccinate lyase, but in the proximal convoluted tubule, synthesis is the highest, which is consistent to the highest expression of arginosuccinate synthetase and arginosuccinate lyase in the nephron (Levillain et al, 1990; Levillain, 2012). l-Arginine synthesis gradually decreases in the terminal part in the outer medulla, ie, in the proximal convoluted tubule, where lower but significant synthesis takes place.

Notably, approximately 83% of l-citrulline released from the small intestine undergoes renal metabolism. Thus, circulating l-citrulline availability is the limiting factor in renal l-arginine production (Windmueller and Spaeth, 1981; Dhanakoti et al, 1990). At least in rats, endogenous production of l-arginine from l-citrulline is necessary for normal growth, and it cannot be completely restored by the diet, demonstrating the importance of renal production of l-arginine for optimal growth in young animals (Hoogenraad et al, 1985). Surprisingly, in rats, the level of l-arginine in plasma is normally stable even after chronic renal failure due to the increased plasma concentration of l-citrulline and the increase in urea, which may inhibit the arginase activity (Moradi et al, 2006).

The total arginase activity is low in the kidney, and its function is still not fully understood. The predominant arginase isoform expressed in the kidney is the mitochondrial isoenzyme Arg2, which is also known as “the kidney arginase.” It is constitutively expressed in the kidneys of humans, rodents, and likely in all mammals, whereas Arg1 is not expressed in the kidney under homeostatic conditions (Spector et al, 1983; Morris et al, 1997; Miyanaka et al, 1998; Morris et al, 2011). In rats, Arg2 is expressed mostly in the proximal straight tubule (Miyanaka et al, 1998). Studies performed on male and female mice showed that female mice have 3-fold higher Arg2 expression and activity compared to male mice (Levillain et al, 2005a). In rats, Arg2 is expressed at higher levels in the inner medullary collecting ducts as compared to the thin descending and ascending limbs of Henle’s loop. Accordingly, the enzymatic activity of Arg2 is not homogenous in the entire kidney, but it occurs mainly in the outer stripe of the outer medulla and inner medulla (Levillain et al, 1989).

Interestingly, there is evidence that all 3 NOS isoforms are expressed in the inner medullary collecting duct (as summarized previously by LoBue et al, 2023), which may indicate a mutual regulation between these 2 enzyme classes in the metabolism of l-arginine in the kidney (Wu et al, 1999; Levillain et al, 2005b; Hyndman et al, 2013). It has been hypothesized that renal arginase activity may be important for l-ornithine production and subsequently polyamine metabolism for the maintenance of normal tissue homeostasis and, only in a small part, for producing urea, which may contribute to concentrating the urine in the medulla (Levillain et al, 1989; Waddington et al, 1998; Brosnan and Brosnan, 2004).

Interestingly, diabetic *Arg2*^*−/−*^ mice chronically treated with streptozotocin were protected against diabetic nephropathy. The lack of Arg2 in these mice protected them against streptozotocin-induced albuminuria, macrophage recruitment, and histopathological changes, leading to renal tissue protection and thus suggesting a role of Arg2 in diabetic nephropathy. Moreover, the lack of Arg2 protection lead to a decrease in renal modular blood flow, which is consistent with preserved renal NO production (Morris et al, 2011). Accordingly, it was shown that a specific lack of Arg2 in ECs reduced renal fibrosis in mice by restoring NO levels and mitochondrial function in the kidney (Wetzel et al, 2020). Another interesting study indicates that Arg2 plays a role in the circadian clock. In this study, it was shown that the lack of Bmal1 in the nephron led to increased urea levels in the plasma, which was correlated with tubular dysfunction (Nikolaeva et al, 2016). The increase in urea were associated to an increase in the activity of Arg2 in the kidney.

Unlike Arg2, Arg1 expression in the kidney occurs only in pathological conditions, mainly as a consequence of inflammation or tissue damage. For example, in nephritic glomeruli in rats, arginase activity was found to be 6-fold higher as compared to control glomeruli; the increased arginase activity was due to the induction of Arg1 expression, whereas Arg2 was not upregulated (Waddington et al, 1998). The expression of Arg1 is likely due to the presence of infiltrating macrophages in the tissue. In fact, high expression of Arg1 was also found in the macrophages located in the outer medulla after I/R injury in mice (Shin et al, 2022). In this study, the authors proposed that Arg1 activity may contribute to stimulating the reparative proliferative response to replace the cells in the medullary tubule.

To summarize, the kidney is a key organ involved in the synthesis of l-arginine and the maintenance of l-arginine levels in plasma. Although expression of Arg2 is constitutive in kidney cells, its function is not fully understood. The main role of Arg2 is likely to keep normal tissue structure/homeostasis during the production of l-ornithine and the subsequent metabolism of polyamines; however, this needs to be further investigated. In contrast, the expression of Arg1 is mainly induced during tissue damage and inflammation and contributes to immunomodulation and tissue repair.

### G Summary and outlook

In summary, Arg1 and Arg2 play multiple, often species-specific roles across cells and tissues in the body. In this context, recent studies carried out with cell-specific transgenic mouse models are providing new and somewhat unexpected information on the biological roles of arginase in specific cells and compartments. Accumulating evidence also indicates that there are important species-specific differences, in particular between rodents and humans, regarding the role of arginases in immune cells and RBCs. These differences need to be considered in pharmacological and translational studies, as well as in preclinical testing.

In the liver, the cytosolic isoenzyme Arg1 is crucial for detoxifying ammonia via the urea cycle, while the role of mitochondrial Arg2 appears to be mainly the synthesis of l-ornithine as a precursor of l-proline and polyamines.

In the immune system, Arg1 is well known to modulate immune cell function and promote immunosuppression, host protection, and resolution of inflammation, mainly by limiting l-arginine bioavailability for downstream metabolic pathways and iNOS-mediated high-output NO synthesis. In mice, arginase expression in M2 macrophages drives the anti-inflammatory responses, as well as participates in tissue repair via polyamine and proline synthesis. In humans, Arg1 is constitutively expressed in granules of PMN cells and MDSCs, and its l-arginine-depleting activity drives immunosuppression of T cell responses. The expression and function of Arg1 in monocytes/macrophages in humans is still debated. The immunosuppressive role of arginases is part of the pathophysiology of chronic inflammatory conditions, infections, and cancer; for example, some parasites and tumor cells express their own arginase or induce arginase expression in the cells of the host. In addition, Arg2 is emerging as a further important regulator of the immune response and a possible pharmacological target.

In the blood, Arg1 is also present at higher levels in human and primate RBCs, whereas its presence in the RBCs of other mammals including mice and rats is very low. In humans, arginase activity is increased in RBCs of people with SCD, thalassemia, and other hemoglobinopathies, and it was shown to promote endothelial dysfunction and pulmonary hypertension by hemolysis-induced arginase release into the plasma and systemic l-arginine depletion. High levels of RBC arginase are also found in people with CVD and diabetes and are proposed to contribute to endothelial dysfunction and cardiovascular events. Interestingly, an immunosuppressive function of arginase release from RBCs has been also proposed.

In the vasculature, arginase activity is increased under pathological conditions such as diabetes and atherosclerosis and promotes endothelial dysfunction, mainly by competing with eNOS for l-arginine and affecting endothelial NO production. The specific role of cytoplasmic Arg1 or mitochondrial Arg2 in the vessel wall is not fully understood. Mice lacking EC Arg1 show no changes in vascular endothelial function ex vivo or in vivo under homeostatic conditions.

In the kidney, Arg2 is likely involved in l-arginine metabolism into l-ornithine and polyamines. It is upregulated and exerts a protective function on the kidney tissue in diabetic nephropathies, at least in the mouse.

These findings underscore the need for further research to reveal the complex roles of Arg1 and Arg2, by considering their cellular and subcellular localization and regulation, their species specificity, and their significance in cellular processes. These differences need to be taken into consideration when pharmacological therapies are tested in preclinical models.

## V l-Arginine metabolism and arginase activity in human disease

There are multiple human studies investigating l-arginine metabolism in human cohorts. l-Arginine levels and metabolism in humans have been extensively studied to investigate the role of arginase in the urea cycle and the consequences of genetic defects (such as hyperarginemia) in liver homeostasis, as well as in the immune system and cancer cells in relationship to iNOS activity. Moreover, l-arginine bioavailability has been studied in the context of arginase as a counterpart of eNOS for endothelial dysfunction in CAD and diabetes. Arginase has shown potential as a therapeutic target for various diseases and conditions, and its inhibition has been explored in clinical trials to evaluate its efficacy and safety. Details about the human studies discussed in the text are summarized in Table 5.

**Table 5:** Summary of human studies on arginases and their substrates and products

Study Subjects Cohort (Number of Subjects)	Parameters	Intervention	Main Findings	Reference
**Measurement of l-Arginine Bioavailability in Human Cohorts**				

Subjects without significantly obstructive CAD (402) vs subjects with significantly obstructive CAD (608)	GABR in plasma	n.a.	• GABR ↓ and L-citrulline level ↑ • Associated with the development of significantly obstructive atherosclerotic CAD and increased the risk of MACE	Tang et al, 2009
CAD (2236)	GABR, l-arginine-to-l-ornithine ratio in serum	n.a.	• GABR inversely correlated with endothelial pro-inflammatory markers such as ICAM-1 and VCAM-1 • Decrease in GABR and arginine-to-ornithine ratio are associated with a significant increase in cardiovascular mortality • GABR ↓ in subjects with T2DM vs subjects without diabetes	Sourij et al, 2011
T2DM (41)	GABR In plasma	Intensified risk factor intervention therapy	• GABR and l-arginine-to-l-ornithine ratio ↑ after 3 mo of intensified risk factor intervention (antihyperglycemic, anti-hypertensive, and antihyperlipidemic therapy)	Tripolt et al, 2012
Adults with COVID-19 (32) and children with COVID-19/MIS-C (20) vs adult controls (28)	GABR, l-arginine-to-l-ornithine ratio in plasma	n.a.	• l-arginine↓,l-arginine-to-l-ornithine ratio↓, and GABR ↓ in the COVID-19−positive adult and COVID-19/MIS-C pediatric group vs control group • Low GABR associated with immune dysregulation and endothelial dysfunction in COVID-19 • Low l-arginine-to-l-ornithine ratio associated with an elevated arginase activity	Rees et al, 2021
Subjects with STEMI (70)	l-arginine metabolite levels in plasma	NOS inhibitor, l-NAME	• Median concentration of l-arginine in acute phase of myocardial infarction >6-mo follow-up measurements correlated with the area at risk and infarct size • Median l-citrulline/l-arginine ↓, l-citrulline/l-ornithine and arginine/ADMA = pointing to a shift of l-arginine metabolism from NOS toward arginase • Low l-arginine concentration associated with worse long-term outcomes	Molek et al, 2021

**Arginase Expression and Activity in Human Disease**				

Early phase of MI (100)	Arginase activity in serum after myocardial infarction (measured from a few hours after the first attack of coronary pain until 5 days)	n.a.	• Arginase activity ↑ in the 10–30 h after MI • Normal values after 3–5 days • No changes in individuals with angina pectoris, acute coronary insufficiency, left cardiac failure, right cardiac failure, or cardiac insufficiency	Porembska and Kedra, 1975
PAH (41) vs controls (37)	l -arginine metabolites, arginase activity in pulmonary artery ECs	n.a.	• Arginase activity ↓ and Arg2 expression ↓ in pulmonary artery ECs from the lung of individuals with PAH	Xu et al, 2004
SCD (228) vs controls (36)	Amino acid levels (Arg, Orn, Cit, and Pro) and arginase activity in plasma, pulmonary hypertension, mortality	n.a.	• Plasma arginase activity ↑ • Correlation between arginase activity and l-arginine-to-l-ornithine ratio • Correlation between arginase activity and increased intravascular hemolytic rate • Low l-arginine-to-l-ornithine ratio associated with greater severity of pulmonary hypertension and mortality	Morris et al, 2005a
Thalassemia (14) vs controls (36)	Amino acid levels (Arg, Orn, Cit, and Pro) and arginase activity in plasma	n.a.	• l-arginine levels ↓,l-ornithine levels ↑, l-citrulline ↑ • l-arginine-to-l-ornithine ratio ↓ • arginase activity in plasma ↑	Morris et al, 2005b
SCD (35) vs controls (10)	Arginase and NOS activity in plasma and RBCs, fetal hemoglobin levels blood count	23 participants with SCD with HU therapy, 12 participants with SCD without HU therapy	• Arginase activity ↓ in individuals with HU therapy, a treatment with ribonucleotide reductase inhibitor • Fetal hemoglobin levels ↑ NOS activity ↑in subjects with HU therapy	Iyamu et al, 2005
Asthma (6) vs controls (7)	Arg1 expression in lung tissue	n.a.	• Arg1 expression ↑ in subjects with asthma	North et al, 2009
MI (43) vs controls (33)	Arg1 activity and expression in serum, L-arginine, and ADMA concentrations in plasma	n.a.	• Arginase activity ↓ and arginase expression ↑ in blood serum from people with MI • Arginase expression negatively associated with left ventricular ejection fraction • Low l-arginine/ADMA ratio in plasma of participants with MI	Bekpinar et al, 2011
Endothelial dysfunction (110) vs controls (106)	Arg1 and Arg2 in plasma (levels and activities)	n.a.	• Arg1 genetic variations affect ED severity • Arg2 concentrations ↑ in plasma of people with ED	Lacchini et al, 2015
Asymptomatic HIV (19), AIDS (33) vs lymph node controls (13), peripheral blood controls (20)	Arg1 expression in lymph nodes and peripheral blood	n.a.	• Arg1 expression ↑ in the lymph nodes from HIV-infected participants	Zhang et al, 2016
Asthmatics (52) vs controls (51)	Arginase activity in serum, expression of Arg2 in airway epithelium	n.a.	• Arg2 expression ↑in the airway of asthmatic study participants	Xu et al, 2017
T2DM (46) vs controls (34)	Arginase activity in RBCs forearm blood flow, RBC (human)-aorta (rat) coincubation	Incubation of RBCs with ABH ex vivo	• Arginase activity in RBCs from subjects with T2DM ↑ • RBC from subjects with T2DM-induced endothelial dysfunction • Inhibition of ROS and arginase prevented endothelial dysfunction in ex vivo bioassay	Zhou et al, 2018
T2DM (27) vs controls (23)	Arginase expression and activity in RBCs	Effects of glucose on RBC arginase activity ex vivo, effects of RBC on I/R in Langendorff heart bioassay	• RBC arginase activity ↑ and production of ROS ↑ in RBCs • RBCs from participants with T2DM aggravate myocardial I/R injury in Langendorff heart • Inhibition of arginase in RBCs improves postischemic myocardial recovery	Yang et al, 2018
T2DM (18) vs controls (20)	RBC (individuals)-aorta (rat) coincubation, arginase activity in aortic rings	n.a.	• Peroxynitrite scavenging with FeTTPS in RBCs reversed endothelial dysfunction in bioassay ex vivo • Upregulation of arginase in RBCs of participants with T2DM and vasculature is peroxynitrite-dependent	Mahdi et al, 2020b

**Administration of Arginase Inhibitors**				

CAD (16), CAD and T2DM (16) vs controls (16)	Arginase expression in the arteries, EDV	nor-NOHA	• Inhibition of arginase significantly improves endothelial function in participants with CAD and T2DM • Upregulation of arginase activity is a critical factor in endothelial dysfunction	Shemyakin et al, 2012
CAD (12) vs CAD and T2DM (12)	EDV	nor-NOHA	• Inhibition arginase provides protection against I/R-induced endothelial dysfunction in participants with CAD	Kövamees et al, 2014
CAD (16), CAD and T2DM (16) vs controls (16)	EDV	nor-NOHA	• Inhibition of arginase improves microvascular endothelial function in humans with T2DM and microvascular dysfunction • Inhibition of arginase protects against I/R-induced endothelial dysfunction in humans with CAD	Kövamees et al, 2016a, Mahdi et al, 2018)
Controls (21)	EDV	nor-NOHA	• Baseline EDV inversely associated with the age of the participants • Inhibition of arginase improves EDV, associated with the age of the participants • Inhibition of arginase improves endothelial function in elderly healthy subjects, age-dependent	Mahdi et al, 2019

**Supplementation of****l****-Arginine/****l****-Citrulline**				

Subjects referred to tertiary treatment for heart failure	Forearm blood flow, 6-min walk test, symptom scores	l-arginine	• Supplementation of l-arginine significantly increased the forearm blood flow during forearm exercises, the waking distance in 6-min walking test, and lowered symptom scores	Rector et al, 1996
Healthy individuals (26)	Forearm resistance arteries, major amino acids in plasma	28-day l-arginine	• l-Arginine supplementation had no effect on endothelial function in healthy adults, induced changes in the total amino acid profile but not l-arginine concentration in plasma	Chin-Dusting et al, 1996
Subjects with chest pain and coronary endothelial dysfunction	Coronary blood flow	6-mo l-arginine	• Long-term supplementation of l-arginine increased coronary blood flow, is associated with improved symptom scores and with a decrease in plasma endothelin concentrations	Lerman et al, 1998
T2DM (25)	Arginase activity in plasma; levels of nitrite and nitrate in plasma	Supplementation of l-citrulline	• Supplementation of l-citrulline reduced arginase activity and plasma NO levels in individuals with T2DM	Shatanawi et al, 2020

**Treatment with PEG-Arginase**				

Arginase auxotrophic tumor (23)	l-arginine level in plasma, PEG-BCT-100 level in plasma, change in tumor size	Intravenous PEG-BCT-100	• Median l-arginine concentration in plasma reached 2.5 μM after the second PEG-BCT-100 injection • Preliminary antitumor activity in 4 cases	Cheng et al, 2021
Arg1 deficiency (32)	l-arginine level in plasma, functional mobility (Gross Motor Function Measure part E and 2-min walk test)	Intravenously/subcutaneously, once-a-week pegzilarginase treatment	• Pegzilarginase treatment lowered mean l-arginine in plasma from 354.0 *μ*M to 86.4 *μ*M as compared to patients treated with placebo from 464.7 *μ*M to 426.6 *μ*M • Patients treated with pegzilarginase showed clinically relevant functional mobility improvements	Russo et al, 2024

ABH, 2(*S*)-amino-6-boronohexanoic acid; ED, erectile dysfunction; EDV, endothelium-dependent vasodilation; FeTTPS, 5,10,15,20-tetrakis(4-sulfonatophenyl) porphyrinato iron III chloride; HU, hydroxyurea; ICAM, intercellular adhesion molecule; l-NAME, *N*^G^-nitro-l-arginine methyl ester; MACE, major adverse cardiovascular event; MI, myocardial infarction; MIS-C, multisystem inflammatory syndrome in children; n.a., not applicable; PAH, pulmonary arterial hypertension; PEG, polyethylene glycol; PEG-BCT-100, pegylated recombinant human arginase 1 BioCancerTreatement international (BCT)-100 (NCI cancer tesaurus code 88286); Pegzilarginase, recombinant, cobalt-substituted and pegylated human ARG1 enzyme (Stone et al, 2010); ROS, reactive oxygen species; STEMI, ST elevation myocardial infarction; VCAM, vascular cell adhesion molecule.

### A Measurements of arginase expression and activity in human disease

The main pathophysiological consequence of autosomal *ARG1* mutations in humans is hyperargininemia, which leads to an autosomal inborn error in the urea cycle (Diez-Fernandez et al, 2018). Other symptoms are progressive intellectual impairment and neurological impairment, persistent growth retardation, and spastic paraparesis (Diez-Fernandez et al, 2018). In a study, 66 mutations of the *ARG1* gene were identified in 112 humans with hyperargininemia; 30 were missense mutations, 15 deletions, 10 splicing, 7 nonsense, 1 small insertion, and 1 translation initiation codon mutation. The estimated incidence of this disease is approximately 1:726,000 (Catsburg et al, 2022). At the beginning of this year, pegzilarginase, a recombinant, cobalt-substituted, and pegylated human ARG1 enzyme therapy, received approval as an orphan drug in the European Union for the treatment of Arg1 deficiency (Russo et al, 2024). Interestingly, mutations of the *ARG1* gene could also play a role in other diseases. In subjects with erectile dysfunction (n = 110), 2 different polymorphisms in the *ARG1* gene were associated with the severity of erectile dysfunction, but there was no correlation with plasma Arg1 levels (Lacchini et al, 2015).

It was proposed that an upregulation of Arg1 in the lungs leads to an imbalance in l-arginine/NO availability, resulting in pulmonary hypertension and/or smooth muscle contraction as well as lung tissue remodeling. In fact, an upregulation of Arg1 has been found in pulmonary diseases, including chronic obstructive pulmonary disease, pulmonary hypertension, pulmonary fibrosis, tuberculosis, and asthma (North et al, 2009; Henno et al, 2015; Monin et al, 2015; Lucca et al, 2018; Wu et al, 2019).

Increased levels of Arg1 in human RBCs and plasma have been proposed to contribute to the pathophysiology of hemoglobinopathies such as SCD and thalassemia. It has been shown that arginase activity in plasma is higher and l-arginine plasma level is lower in people with thalassemia (n = 14) or SCD (n = 140) (Morris et al, 2005a,b). The authors of these elegant studies proposed that intravascular hemolysis with release of hemoglobin and arginase in plasma causes, on one hand, a reduction in l-arginine concentration in plasma, and on the other hand, scavenging of NO, leading to endothelial dysfunction and pulmonary hypertension. Interestingly, the treatment of humans with SCD with hydroxyurea (n = 23) reduced arginase activity in the plasma (Iyamu et al, 2005).

In addition, the first evidence that Arg1 present in RBCs can modulate endothelial dysfunction and the outcome of I/R injury has come from bioassays (Yang et al, 2018; Zhou et al, 2018; Mahdi et al, 2020b). RBCs from people with T2DM (n = 20) showed increased arginase activity and arginase level in RBCs as compared to healthy individuals (n = 15) (Zhou et al, 2018). Furthermore, the authors showed in a bioassay that the coincubation of RBCs from individuals with T2DM with rat aortas induces endothelial dysfunction, which can be prevented by the ex vivo inhibition of arginase. In addition, RBCs from people with T2DM also induced an increase in arginase activity in coincubated human carotid arterial ECs. The authors of the study proposed that this upregulation is induced by peroxynitrite (Mahdi et al, 2020b). In another study, the same authors showed that RBCs from people with T2DM (n = 13) aggravate myocardial I/R injury (Yang et al, 2018). These studies indicate that Arg1 present in human RBCs plays a role in the complex vascular and cardiac pathophysiological consequences of T2DM in humans.

There are multiple studies showing increased Arg1 activity/protein levels in serum of humans with myocardial infarction (Porembska and Kedra, 1975; Bekpinar et al, 2011), and they were linked to endothelial dysfunction via decrease of l-arginine bioavailability.

An interesting hypothesis that should be also taken into consideration is that the release of arginase and decrease of endogenous levels of l-arginine may also exert an immunosuppressive effect, as hypothesized by Munder (2009). The immunosuppression may become pathophysiological in various infections with parasites and viral infections. For example, in human immunodeficiency virus (HIV)-infected humans, a high expression of Arg1 in lymph nodes correlated with an increase in HIV viral load whereas iNOS expression negatively correlated with the HIV viral load (n = 52) (Zhang et al, 2016). Such upregulation may contribute to the suppression of antiviral immunity in HIV-infected humans; thus, Arg1 expression can be used as a parameter to predict disease progression.

There are no known genetic defects of Arg2 that cause human disease. However, it has been shown that Arg2 expression and activity were increased in pulmonary artery ECs of individuals with pulmonary arterial hypertension (n = 41 ± 3) (Xu et al, 2004). Similar to Arg1, Arg2 expression is increased in people with asthma caused by chronic airway inflammation (Xu et al, 2017).

In summary, both arginase isoforms play crucial roles in various disease conditions. Mutations in the *ARG1* gene lead to a defect of the urea cycle, causing hyperargininemia and leading to early death. In the beginning of 2024, the first therapy using human recombinant pegylated cobalt-substituted arginase enzyme became available. On the other hand, the upregulation of arginase expression and activity seems to be involved in various diseases such as T2DM and SCD. In addition, arginase shows immunosuppressive properties, which are involved in infections with parasites and viral infection.

### B Measurement of l-arginine bioavailability in human cohorts with cardiometabolic disease

The majority of the human studies having l-arginine bioavailability or metabolism as a primary outcome measure is based on the hypothesis that arginase activity in the vasculature contributes to the consumption of l-arginine, which contributes to endothelial dysfunction. Consumption of l-arginine and the production of l-ornithine should affect GABR in plasma, as an index of l-arginine bioavailability. Therefore, GABR was measured in the plasma of people with CAD (those without significantly obstructive CAD, n = 402 vs significantly obstructive CAD, n = 608) by monitoring the levels of free l-arginine, l-ornithine, l-citrulline, and ADMA. GABR was lower in participants with obstructive CAD (>50% stenosis) than in those without obstructive CAD, which implied that decreased GABR is associated with obstructive CAD and a subsequent increased incidence of major adverse cardiovascular events (Tang et al, 2009). Similarly, the levels of l-arginine, l-ornithine, and l-citrulline were measured in the serum of participants (n = 2236) collected before coronary angiography (Sourij et al, 2011). In this study, they found that GABR was significantly decreased in persons with T2DM and was also inversely correlated with some known biochemical markers of endothelial dysfunction such as the expression of intracellular adhesion molecule-1, vascular adhesion molecule-1, and von Willebrand factor. Furthermore, they also observed that decreased GABR and the arginine-to-ornithine ratio were associated with increased cardiovascular mortality. A further study investigated the changes of GABR in people with T2DM (n = 41) after intensifying the therapy according to the guideline for 3 months to reach therapy targets such as hemoglobin A_1c_, low-density lipoprotein cholesterol 2.6, or blood pressure (Tripolt et al, 2012). The authors showed that targeting those risk factors improves GABR and arginine-to-ornithine ratio. It is important to note that this improvement was only found in people who had T2DM for <5 years.

l-Arginine levels and metabolism were also measured in plasma samples from subjects during the acute phase of myocardial infarction collected before percutaneous coronary intervention and again 6 months after myocardial infarction (n = 70) (Molek et al, 2021). l-arginine, l-ornithine, and ADMA levels were determined and calculated into indexes of l-citrulline/l-arginine, l-citrulline/l-ornithine, and l-arginine/ADMA. The authors observed that the index of l-citrulline/l-arginine significantly decreased in the acute phase of myocardial infarction, but the indexes of l-citrulline/l-ornithine and l-arginine/ADMA were unchanged. The authors proposed that this may indicate a shift of l-arginine utilization from NOS toward arginase and/or an increase in the activity of arginase.

l-arginine and l-ornithine concentrations were also measured in the plasma from lean and obese people with asthma, obese people without asthma, and corresponding healthy controls using liquid chromatography-MS/MS (Winnica et al, 2019). In this study, they demonstrated that the l-arginine levels in the plasma were decreased in both lean and obese people with asthma, and also obese people without asthma as compared with that of healthy individuals. Moreover, the l-arginine levels in obese people with asthma were significantly lower than those in healthy lean controls.

Recently, l-arginine level, l-arginine-to-l-ornithine ratio, and GABR were analyzed in adults diagnosed with COVID-19 (n = 32) and children with COVID-19/multisystem inflammatory syndrome (n = 20). They found that all these parameters were significantly lower in COVID-19–positive adults and COVID-19/MIS-C children than in COVID-19-negative controls. The authors proposed that low arginine-to-ornithine ratio in these people might be due to elevated arginase activity and that low GABR may contribute to immune dysregulation and endothelial dysfunction in COVID-19 (Rees et al, 2021).

### C Arginase as a pharmacological target

Depending on the pathophysiological role of arginase and the specific type of disease, targeting arginase involves 2 possible strategies including arginase-mediated l-arginine depletion or inhibition of arginase to restore l-arginine bioavailability. Established and potential therapeutic applications are listed in Table 3.

Administration of pegylated recombinant arginase has been recently approved by the European Medicines Agency to treat humans with genetic hyperarginemia. Moreover, pegylated arginase has been proposed as an antitumor agent (similar to the antileukemia agent l-asparaginase) for tumors susceptible to l-arginine deprivation, such as tumors lacking argininosuccinate synthetase enzymes (Cheng et al, 2021). If properly targeted to the site of inflammation, another possible application of recombinant Arg1 may be immunosuppression in autoimmunity and unwanted inflammatory reactions (Munder, 2009).

Applications of arginase inhibitors were proposed for diseases with pathological upregulation of arginase such as specific infections, cancer, or endothelial dysfunction in T2DM and CAD, and to treat pulmonary hypertension in SCD and hemoglobinopathy (see Section V.A).

There are 3 generations of arginase inhibitors, which are summarized in Table 4. These inhibitors do not show isoform specificity, need to be administrated systemically via intraperitoneal or intravenous injection, have a short half-life, and are rapidly eliminated by the kidney. A well known and often-used arginase inhibitor in cell culture and animal studies, and also in human studies is nor-NOHA. Nor-NOHA is a derivate of NOHA, a stable intermediate of NO synthesis by NOS. It is a competitive nonspecific inhibitor of arginase and is considered as one of the most potent arginase inhibitors (Colleluori and Ash, 2001; Pudlo et al, 2017). Numerous studies have investigated the effect of arginase inhibitors in animals (Kim et al, 2009; Jung et al, 2010; El-Bassossy et al, 2013; You et al, 2013; Pera et al, 2014), and recently, in humans (Table 5).

There are very promising studies showing that arginase inhibition by nor-NOHA improves endothelium-dependent vasodilation (Shemyakin et al, 2012; Kövamees et al, 2014, 2016a; Mahdi et al, 2018). In human studies, nor-NOHA (0.1 mg/min for 2 hours) was administrated by infusion (Shemyakin et al, 2012, Kövamees et al, 2014, 2016a; Mahdi et al, 2019). Administration of nor-NOHA improved endothelial function in healthy elderly humans (n = 21) as determined by forearm venous-occlusion plethysmography (Mahdi et al, 2019).

Recently, a third generation of arginase inhibitors was developed and tested (Table 4). One notable compound from this generation is INCB001158 (formerly CB1158), an oral arginase inhibitor that exhibits higher specificity for Arg1 (IC_50_ = 86 nm) than Arg2 (IC_50_ = 296 nm). INCB001158 has been evaluated in phase 1/2 clinical trials for the treatment of solid tumors with increased arginase activity in combination with chemotherapy (Steggerda et al, 2017; Kuboki et al, 2024; Naing et al, 2024). The most notable result of these studies is that arginase inhibitors may be effective for therapies of specific tumors with upregulation of arginase activity as a mechanism for l-arginine depletion and immunosuppression.

In conclusion, pegylated arginase is an approved drug for hyperarginemia and genetic defect and potentially for l-arginine auxotrophic tumors susceptible to l-arginine deprivation and for immunosuppression.

The clinical applications of arginase inhibitors have shown potential to improve endothelial function and vasodilation in individuals with T2DM, enhancing the effects of l-arginine supplementation in individuals with heart failure and CADs and serving as a promising therapeutic target for therapy of specific cancers. Further pharmacological research focused on developing and testing arginase inhibitors will contribute to the development of novel treatments and therapies in the future.

### D l-Arginine/l-arginine metabolite supplementation

Oral supplementation of l-arginine has been proposed as a way to increase l-arginine bioavailability and to boost production of NOS-derived NO in humans (Girerd et al, 1990). For example, l-arginine supplementation has been proposed to alleviate endothelial dysfunction (Lerman et al, 1998; Bai et al, 2009). However, the results of these studies are controversial.

Oral l-arginine intake was shown to positively affect people with heart failure and peripheral artery occlusive disease by increasing the distance in the 6-minute walk test, increasing forearm blood flow during forearm exercise, or reducing the symptom score significantly (Rector et al, 1996; Lerman et al, 1998). On the other hand, oral administration of l-arginine in healthy people (n = 26) did not improve systemic hemodynamics in vivo or vascular function of gluteal subcutaneous arteries assessed in vitro (Chin-Dusting et al, 1996).

### E Summary and outlook

The involvement of arginases in the urea cycle, liver function, immune response, and cancer cell dynamics, particularly in relation to iNOS activity, has been extensively examined. In addition, arginase activity in the vessel wall has been studied in the context of endothelial dysfunction in CAD and diabetes, revealing its potential as a therapeutic target. GABR is a measure of l-arginine bioavailability and has been found to be lower in people with obstructive CAD and T2DM, correlating with major adverse cardiovascular events and endothelial dysfunction markers. Pharmacological interventions that improve risk factors for T2DM have been shown to improve GABR, particularly in people recently diagnosed with diabetes. Mutations in the *ARG1* gene cause hyperargininemia, leading to various health complications. Arginase activity is also increased in several pulmonary diseases and hemolytic anemias, affecting NO availability and contributing to disease pathology. The arginase inhibitor nor-NOHA has demonstrated efficacy in improving endothelial function in people with T2DM and healthy elderly individuals. Supplementation with l-arginine or l-citrulline has been explored to enhance l-arginine bioavailability and NO production, with varying results depending on the health status of individuals.

## VI Conclusion and outlook

Arginases exhibit diverse functions across various tissues. In the liver, Arg1 is indispensable for ammonia detoxification, while the specific role of mitochondrial Arg2 needs further investigation. It is important to point out that there are significant differences in Arg1 and Arg2 expression, cellular localization, and activity in humans and primates as compared to rodents (mice, rats) and other mammals, in particular in the blood and bone marrow, and vascular ECs.

In the immune system, the distribution and function of Arg1 are very different in mouse and man. While in mice, blood Arg1 is mainly expressed in myeloid cells and is upregulated by Th2 cytokines, in humans, Arg1 is constitutively expressed in PMN cells and is not regulated by Th2 cytokines. In both species, arginase activity has an anti-inflammatory and immunosuppressive effect on T cells.

In human and primate RBCs and proerythroblasts, Arg1 expression and activity are high, while in rats, mice, and other mammals, it is very low and/or barely detectable. In humans, elevated arginase activity in RBCs from individuals with SCD or hemoglobinopathies suggests a contributory role in disease pathogenesis, with similar observations in CVD and diabetes, underlining its fundamental importance in human disease.

In CVD conditions such as diabetes and atherosclerosis, increased arginase activity can lead to endothelial dysfunction by competing with eNOS for the bioavailability of l-arginine, thus impairing NO production and leading to endothelial dysfunction. Accordingly, administration of arginase inhibitors in people with T2DM improves vascular function. Surprisingly, EC-specific *Arg1*^*−/−*^ mice showed no changes in endothelial function or cardiovascular hemodynamics, but global *Arg2*^*−/−*^ mice showed hypertension, indicating that at least in vivo*,* the role of vascular Arg1 in maintaining endothelial function under homeostatic conditions in mice is limited.

Although the kidney plays a major role in the control of l-arginine synthesis, systemic levels and bioavailability, the role of arginases is the kidney is not fully understood. The main isoform expressed is Arg2, and it appears to be crucial for tissue integrity and repair (at least in rodents).

Clinical and translational studies have highlighted the therapeutic potential of targeting arginase/l-arginine metabolism in various diseases. Clearly, the described multiple, complex, species-, cell-, and isoform-specific roles of arginase make pharmacological targeting difficult. The only approved drug is pegylated recombinant arginase for the treatment of people with genetic hyperarginemia. However pegylated arginase was proposed as a possible antitumor therapy for tumors sensitive to l-arginine deprivation. The control of l-arginine bioavailability by arginases, as a key factor in endothelial dysfunction in CVD, has been extensively studied, and arginase is still considered a promising therapeutic target. The use of arginase inhibitors (eg, nor-NOHA) was tested in small cohorts of people with endothelial dysfunction due to CAD or T2DM. Moreover, administration of arginase inhibitors was proposed for blocking circulating arginase in hemoglobinopathies (SCD, thalassemia) and specific infectious diseases. Arginase inhibition has shown efficacy in improving endothelial function in subjects with T2DM and healthy elderly individuals, while l-arginine and l-citrulline supplementation have demonstrated potential benefits for heart failure and peripheral arterial occlusive disease. A strong limitation of the currently available inhibitors is that they do not show isoform specificity, need to be administrated systemically via intraperitoneal or intravenous injection, have a short half-life, and are rapidly eliminated by the kidney. The third generation of arginase inhibitors can be administrated orally, have better pharmacodynamics, and have already been tested for cancer therapy.

More studies are needed to understand the complex biological and species-specific roles of Arg1 and Arg2, their importance in cellular processes, and their potential as therapeutic targets. The new pharmacological cell-specific targeting techniques and the applicability of single-cell analysis for personalized medicine will allow better pharmacological strategies to target Arg1 and Arg2 in humans.

## Conflict of interest

No author has an actual or perceived conflict of interest with the contents of this article.
